# Using IT to Improve Outcomes for Children Living With Cancer (SyMon-SAYS): Protocol for a Single-Institution Waitlist Randomized Controlled Trial

**DOI:** 10.2196/50993

**Published:** 2023-09-08

**Authors:** Jin-Shei Lai, Sally E Jensen, John Devin Peipert, Sandra A Mitchell, Sofia F Garcia, David Cella, Stewart Goldman, Alicia Lenzen

**Affiliations:** 1 Medical Social Sciences Feinberg School of Medicine Northwestern University Chicago, IL United States; 2 Robert H. Lurie Comprehensive Cancer Center Northwestern University Chicago, IL United States; 3 Division of Cancer Control and Population Sciences National Cancer Institute Bethesda, MD United States; 4 Department of Child Health College of Medicine - Phoenix University of Arizona Phoenix, AZ United States; 5 Phoenix Children’s Hospital Phoenix, AZ United States; 6 Division of Hematology, Oncology, Neuro-Oncology & Stem Cell Transplantation Ann & Robert Lurie Children’s Hospital of Chicago Chicago, IL United States

**Keywords:** pediatric, cancer, symptom monitoring, randomized controlled trial, protocol, electronic health record, health-related quality of life

## Abstract

**Background:**

Children and adolescents with cancer may experience multiple disease- and treatment-related symptoms that negatively affect health-related quality of life. Routine symptom surveillance thus constitutes an important component of supportive care in pediatric oncology. The Symptom Monitoring and Systematic Assessment and Reporting System in Young Survivors (SyMon-SAYS) system will administer, score, interpret, and display the results of symptom assessments captured weekly using patient-reported outcomes presented via the electronic health record (EHR) portal between clinic visits in oncology ambulatory settings, when patients are likely to be more symptomatic. This study is testing a digital system for routine symptom surveillance that includes EHR-based reports to clinicians and alerts for severe symptoms.

**Objective:**

In this randomized trial, we are examining the effects of the SyMon-SAYS system on perceived barriers to symptom management, self-efficacy, and symptom severity. Better self-management and timely clinical intervention to address symptoms promote adherence to treatment plans, strengthen child and parent self-efficacy, improve interactions between children, parents, and their clinical providers, and optimize clinical outcomes.

**Methods:**

The SyMon-SAYS system is integrated into the EHR to streamline the presentation of symptom scores and delivery of alerts for severe symptoms to clinicians using EHR (Epic) messaging functionalities. Children (aged 8 to 17 years) complete the weekly symptom assessment and review the symptom report by logging into the patient portal (Epic MyChart). This single-institution waitlist randomized controlled trial is recruiting 200 children (aged 8-17 years) with cancer and their parents, guardians, or caregivers. Participating dyads are randomly assigned to receive the intervention over 16 weeks (Group A: 16-week SyMon-SAYS intervention; Group B: 8-week usual care and then an 8-week SyMon-SAYS intervention). Analyses will (1) evaluate the efficacy of SyMon-SAYS at week 8 and the maintenance of those effects at week 16; (2) evaluate factors associated with those efficacy outcomes, including contextual factors, adherence to the SyMon-SAYS intervention, demographic characteristics, and clinical factors; and (3) evaluate predictors of adherence to the SyMon-SAYS intervention and preference of SyMon-SAYS versus usual care.

**Results:**

Data collection is currently in progress. We hypothesize that at 8 weeks, those receiving the SyMon-SAYS intervention will report decreased parent-perceived barriers to managing their children’s symptoms, increased parent and child self-efficacy, decreased child symptom burden, and ultimately better child health-related quality of life, compared to waitlist controls. Feasibility, acceptability, and engagement from the perspectives of the children with cancer, their parents, and their clinicians will be examined using mixed methods.

**Conclusions:**

We anticipate that this system will facilitate prompt identification of problematic symptoms. Additionally, we hypothesize that with the availability of graphical symptom reports over time, and timely provider responses, children or parents will become better informed and take an active role in managing their symptoms, which will further improve clinical outcomes.

**Trial Registration:**

ClinicalTrials.gov NCT04789720; https://clinicaltrials.gov/study/NCT04789720

**International Registered Report Identifier (IRRID):**

DERR1-10.2196/50993

## Introduction

### Background

Cancer can be distressing, in part, due to the unrelieved symptoms caused by aggressive therapy regimens implemented to treat the disease, which can last even after the completion of treatment [1]. Management of cancer-related symptoms in children has not kept pace with these advances in treatments. Children continue to experience distressing symptoms related to cancer and its treatment [2,3], as do childhood cancer survivors at all stages of survivorship. The unrelieved symptoms and side effects of often-aggressive cancer treatments can lead to poor psychosocial functioning and decreased health-related quality of life (HRQoL) for children with cancer and their families [4]. There is a need to improve child or family self-monitoring and self-management skills to better manage symptoms throughout the treatment continuum, from on-therapy to long-term survivorship, in order to provide timely intervention and lessen the adverse impacts of symptoms on HRQoL. Subsequently, it is recommended that children with cancer and their family should receive systematic assessments to identify their psychosocial health care needs [5].

Factors contributing to inadequate symptom management exist at the system, health care provider, and patient levels. Health care system barriers include the structure and organization of care, reimbursement, logistics, and resources [6,7]. Health care provider barriers include limited time available during a patient encounter [8], willingness to elicit information from patients [6,9], and challenges in obtaining systematic symptom assessment [10]. For very young patients, there is the added complexity regarding uncertainty about the accuracy of self-report. Patient-level barriers include failure to report symptoms to clinicians [11], desire to be a “good patient” to avoid conflict [12-14], concern over side effects of prescribed medications [12,15], and a perception that during cancer treatment symptoms are inevitable or untreatable [4]. A patient-centered approach to health care can minimize these barriers. Patient-related factors that facilitate self-management include: proactively seeking information and sharing their opinion about their self-management regimen with providers [16].

Improvements in the quality of communication between patients and health care providers can favorably affect symptom management, including improved information recall [17], satisfaction [18], overall HRQoL [19,20], and adherence to practitioners’ recommendations [21]. According to the IOM consensus report Cancer Care for the Whole Patient: Meeting Psychosocial Health Needs [22], inadequate communication and lack of patient involvement are particularly worrisome, as effective patient-clinician communication is linked to positive health outcomes. For patients and families, information is crucial to promote a sense of control, decrease emotional distress, support effective self-management, and eliminate disruptions to daily activities [23,24]. Research on adult oncology patients showed the average patient asks 5 or fewer questions during a 15-minute doctor’s office visit, with a high proportion asking no questions, suggesting that patients generally are not taking an active role in their care [25]. Previous literature suggests the clinician-patient relationship can be strengthened by the simple addition of a “prompt sheet,” encouraging patients to ask questions about treatment and prognosis; oncologists’ efforts to address the issues raised by the use of prompts promoted patient confidence to ask about prognosis, alleviated patient anxiety and reduced clinic visit length [26]. Yet, most literature examining interventions to strengthen patient-clinician communication about problematic issues has focused on adult patients and there has been limited research on children with cancer. Patients’ developmental ages are critical. Children’s less-developed verbal skills, along with parents’ and clinicians’ possibly conflicting communication styles and attitudes toward children, might prevent children from adequately communicating symptoms to providers [27]. Consequently, children are less likely than adults to discuss their symptoms with health care providers [28].

Health care delivery has shifted from inpatient to outpatient or home settings, leading investigators to focus on integrating technology into symptom monitoring and self-management support. The literature suggests that web-based symptom management programs in patients with pediatric cancer are feasible [29], may improve care, enhance patient and provider satisfaction, and lessen symptom burden, and generally do not increase provider time and effort. Tsimicalis et al [30] reviewed studies incorporating technology in pediatric oncology settings, identifying several benefits including surmounting logistical difficulties of distance, time, costs, and transport [31-34]; improving access to experienced and specialized health care professionals in rural and remote areas [35]; offering support after hours; and avoiding unplanned hospital visits [36]. The scope of research exploring electronic symptom surveillance systems in pediatric oncology has been constrained, primarily concentrating on palliative care [29] and hospitalized children [37]. Both do not capture symptoms that occurred between clinical visits. This study fills a gap in knowledge about the feasibility, acceptability, and effects on self-efficacy and symptom burden of measurement-based symptom care delivered via the electronic health record (EHR) and explores implementation challenges and strategies that will need to be addressed to refine this intervention for optimal efficacy and adoption. The aim of this manuscript is to outline the study protocol (version 4; February 17, 2023) for this waitlist randomized controlled trial.

### Purpose

**Figure 1:**
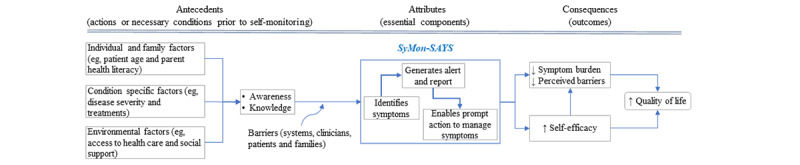
SyMon-SAYS conceptual model modified based on the "Model of the concept of the the self-monitoring" by Wilde and Garvin [38]. SyMon-SAYS: Symptom Monitoring and Systematic Assessment and Reporting System in Young Survivors.

In this trial, we are testing the efficacy of 8 weeks of Symptom Monitoring and Systematic Assessment and Reporting System in Young Survivors (SyMon-SAYS), an EHR-based system for routine symptom surveillance with self-management support to children and parents or caregivers, reports to clinicians and alerts for severe symptoms. The conceptual model of the SyMon-SAYS intervention is shown in Figure 1; the model was modified from a self-monitoring model [38], our SyMon-SAYS pilot study [39], as well as from literature. “Self-monitoring” is defined as awareness of symptoms or bodily sensations that are enhanced through periodic measurements, recordings, and observations to provide information for improved self-management [38]. The interplay among awareness, measurement, and observations can enhance self-management by improving how individuals monitor their health. SyMon-SAYS is designed to capture 2 major attribute components: (1) increased awareness of symptoms, and (2) accurate measurement and recording of symptoms. Adherence to the SyMon-SAYS intervention (ie, completion of weekly reports of symptoms by patients and parents) is hypothesized to be influenced by contextual factors (child or family, condition-specific, environmental factors) as well as barriers and facilitators. Through technology-based monitoring and reporting, the SyMon-SAYS program facilitates self-monitoring of symptoms via systematic symptom assessments and graphic reports based on these assessments and facilitates self-management by directing patients or parents to web-based resources developed by clinicians for appropriate strategies to manage the symptoms identified by the assessment. The resources site can be accessed directly through MyChart. Because the SyMon-SAYS system is designed to help patients and parents track symptoms on a consistent basis, it aims to lessen perceived barriers to managing symptoms and symptom burden, enhance self-efficacy of patients and parents, and ultimately improve HRQoL [23,40-45].

### Objectives and Study Aims

#### Overview

In this single institution modified waitlist control randomized trial, 200 children (aged 8-17 years) with cancer and their parents will participate for 16 weeks (group A: 16-week SyMon-SAYS intervention; Group B: 8-week usual care and then 8-week SyMon-SAYS intervention). Our study is addressing the following specific aims.

#### Aim 1

Evaluate the efficacy of SyMon-SAYS after 8 weeks and its maintenance effects at week 16. We hypothesize that group A (vs group B) will report decreased parent-perceived barriers to managing their children’s symptoms, decreased child symptom burden, increased child and parent self-efficacy, and ultimately increased child HRQoL at week-8. We expect that these differences between Groups A and B will be narrowed at week 16 when Group B completes the 8-week SyMon-SAYS intervention.

#### Aim 2

Evaluate factors associated with aim 1’s efficacy outcomes, including but not limited to (1) contextual factors, (2) adherence to the SyMon-SAYS intervention, and (3) symptom communication between clinicians and children or families.

#### Aim 3

Evaluate predictors of adherence to the SyMon-SAYS intervention and preference of SyMon-SAYS versus usual care. We will identify predictors of adherence and model their association by using ordinal regressions. Preference of SyMon-SAYS versus usual care will be evaluated using responses from SyMon-SAYS program evaluation. This understanding will help to develop individualized symptom management approaches for better quality and outcomes of care in pediatric oncology ambulatory settings.

## Methods

### Ethics Approval

This study was approved by the Institutional Review Boards of Northwestern University (STU00210598) and Ann & Robert Lurie Children’s Hospital of Chicago (IRB 2019-3018). Lurie Children’s maintains records of the authorization and reliance agreements. All subsequent modifications to the protocol will be submitted for review and approval by the Ann & Robert Lurie Children’s Hospital of Chicago. This trial is registered in ClinicalTrials.gov (NCT04789720). The Data and Safety Monitoring Board (DSMB) of the study provides oversight for data and safety monitoring for this trial. The DSMB consists of 3 members outside of Northwestern University and Ann & Robert Lurie Children’s Hospital of Chicago, who have extensive experience in research with clinical trials, patient-centered outcomes, and pediatric oncology. DSMB and the study teams meet annually to review the data and any adverse events that occurred.

### Study Design and Setting

**Figure 2:**
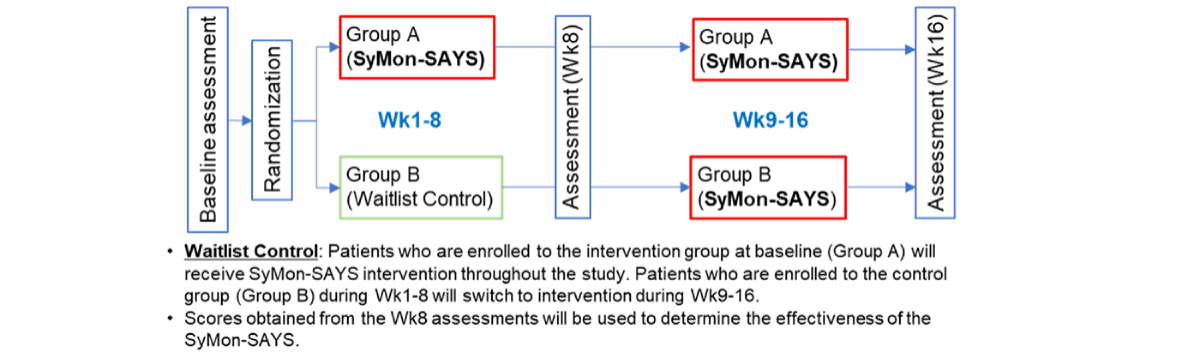
Overall study design. SyMon-SAYS: Symptom Monitoring and Systematic Assessment and Reporting System in Young Survivors. Wk: week.

**Figure 3:**
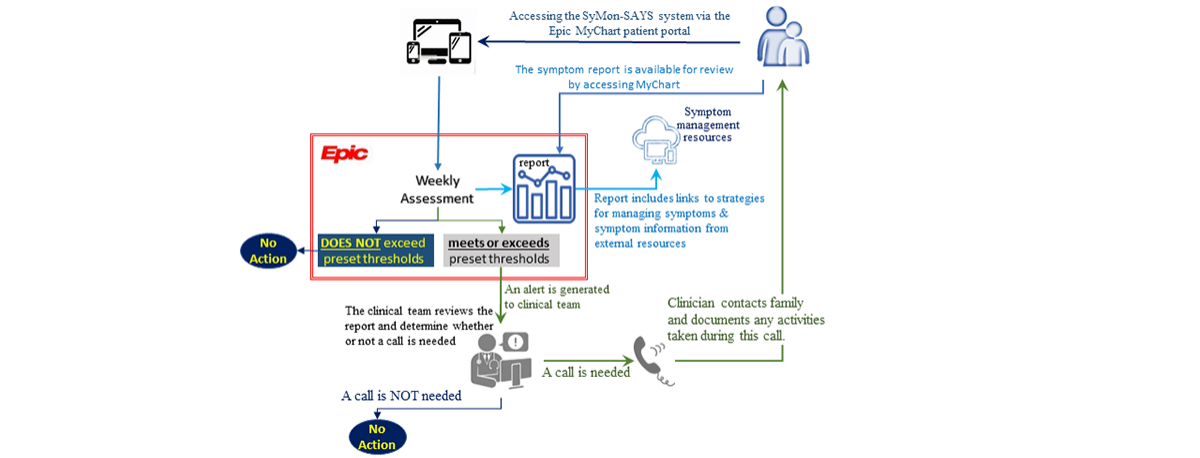
SyMon-SAYS intervention schema. SyMon-SAYS: Symptom Monitoring and Systematic Assessment and Reporting System in Young Survivors.

The study design is shown in Figure 2. To increase the utility and compliance of clinicians, children, and parents, the SyMon-SAYS system was built within the Ann and Robert H. Lurie Children’s Hospital of Chicago (Lurie) EHR (Epic). Participants (parents and children) complete assessments using an iPad or a device with internet access at baseline, week-8, and week-16. They are randomly assigned to Group A (weeks 1-16: SyMon-SAYS intervention) or Group B (weeks 1-8: usual care; weeks 9-16: SyMon-SAYS intervention) after the baseline assessment. During the intervention weeks (either weeks 1-16 or weeks 9-16), participants complete a weekly symptom assessment wherever they have internet access using their MyChart patient portal, accessible via smartphone, tablet, or computer. The SyMon-SAYS intervention schema is shown in Figure 3.

### Participants

#### Overview

We are recruiting 200 children with cancer from hematology, oncology, and brain tumor clinics at Ann & Robert Lurie Children’s Hospital, Chicago with an anticipated 20% attrition rate over a 16-week trial. In order to generalize our results to the entire pediatric cancer population and not exclude important patient subpopulations, we have broad inclusion criteria. Oncology clinical providers who treat participants are also enrolled in the study and provide informed consent as research participants per IRB’s requirements at Lurie Children’s Hospital. Our inclusion criteria are as follows.

#### Patients

Eligible participants are those with a diagnosis of malignancy (including children with primary brain tumors), who are currently receiving cancer-directed therapy or are within 6 months of completing cancer-directed therapy, and who are between 8 and 17 years old at the time of enrollment, English-speaking, have sufficient cognitive and motor abilities to complete surveys via an electronic device (eg, smartphone, iPAD, etc) or computer, and able and willing to sign assent forms (for those between 12-17 years of age). No formal cognitive testing is conducted; eligibility based on cognitive status is determined by the judgment of their clinicians. Usage of any assistive device to complete the assessment is acceptable and will be documented.

#### Parents

A parent of eligible children (father, mother, or legal guardians) is eligible for the study if she or he demonstrates sufficient fluency in English or Spanish to understand and provide informed consent (either in clinic or via remote recruitment), agrees to complete assessments at all time points, and has sufficient cognitive and motor abilities to complete surveys via an electronic device (eg, smartphone, iPAD, etc) or computer. Only one parent per child participates in the study.

#### Clinicians

Eligible clinicians are oncology attending physicians, fellows, nurse practitioners, and nurses who treat participating children at Ann & Robert Lurie Children’s Hospital.

### Informed Consent Procedures

Study personnel explain the aims of the study, what is involved in study participation, and the potential risks and benefits of the study to the potential participants (children, their parents, and providers) so that they can make an informed decision as to whether they wish to participate. This explanation is done in plain, nonexculpatory language that is easy for parents, patients, and providers to understand. Additionally, all participants who are approached for participation in this study are informed that participation in this study is voluntary, and that a decision to not participate will not jeopardize or affect their medical care or their employment status (for clinical providers) in any way. They are informed that they are also free to discontinue participation at any time during the study with no adverse impact on their treatment, overall care, or employment status. Clinical provider participants are also be informed that their decision to participate or discontinue participation will not impact their employment status, and the data they provide during the course of their participation will be used for the purpose of this research study only and will not be made available to the institution or any managing or supervisory personnel (eg, for their merit reviews).

### Risks and Ethical Issues

There are no known social or legal risks to subjects who participate in this study. Our experience with data collection using similar questions has indicated that few (less than 1% of over 15,000 participants over more than 20 years) are distressed by the content of the questions posed to them. Participants are advised that if they become upset by the questions, they will have the option to talk with a trained mental health professional. The principal investigator (PI) will be notified of all requests for consultation with a mental health professional.

There is a slight possibility that enhanced monitoring of symptoms could lead to an increase in anxiety. Yet our feasibility study showed that SyMon-SAYS significantly decreased children’s anxiety levels at week-4 (*P*=.046; unpublished data). Furthermore, in this study, children’s emotional distress (worry and sadness) are included in the weekly assessments and are monitored closely. As with all symptoms being monitored, an alert is generated when children’s reports of worry or sadness meet or exceed the preestablished threshold. Clinicians are notified within 24 hours and take appropriate actions to respond to these symptom experiences. In addition, in consultation with the site clinical team, the study team reserves the right to withdraw any participant for whom there is concern that the intervention is having an adverse effect.

### Measures to Protect Privacy and Confidentiality of Participants

Study personnel enroll patients and their parents and assign each patient and parent participant their unique ID code, which is used for identifying and tracking participants throughout the study. Parent codes are linked to corresponding patient (child or adolescent) codes. Clinician participants are recruited by the study team and are also assigned a unique ID code. All data collected from all participants are linked with this unique identifier and not the participants. Information of linking IDs to participants is saved as a separate password-protected file that only the PI and the study coordinator can access.

### Sample Size Consideration

An effect size of at least 0.33 has been shown to be clinically meaningful in the measurement of PROs in patients with cancer [46,47]. An effect size of 0.45 is relevant when using cancer treatment satisfaction as an end point [48,49]. Therefore, effect sizes between 0.33-0.45 are expected and will represent clinically meaningful differences for the barriers and self-efficacy end points. We anticipate intervention effects will be maintained in Group A over 16 weeks, and comparisons between groups will be made using both week-8 and week-16 data. We have calculated statistical power to detect differences between the study arms at both weeks 8 and 16, taking into consideration attrition. First, we consider a simple 2-group comparison at the primary 8-week follow-up visit for each end point in aim 1. For this comparison, we calculated that a sample size of 180 (90 per group) will provide 80% power to detect effects of 0.42 for the primary comparisons at week 8. Considering effects at week 16, a sample size of 160 (80 per group) will provide 75% power to detect an effect size of 0.42 but would provide 80% power to detect an effect size of 0.45.

Based on our symptom monitoring studies with more than 100 patients with advanced cancer, fewer than 15% of patients dropped out of a 12-week study because of death, disease progression, ineligibility to continue (eg, transfer care or move), or unwillingness to continue study participation. The dropout rate was 9.5% (6/63) in the SyMon-SAYS pilot (8-week). We project a higher dropout rate (40/200, 20%) in the proposed 16-week study period, with approximately half of these dropouts (20/200, 10%) occurring by week 8. To accrue at least 180 patients who complete through week 8, which will provide adequate statistical power outlined above for the primary analyses, we plan to recruit 200 patients at baseline.

### Randomization and Allocation

We will use a stratified block randomization approach. Participants are randomly assigned to Group A or B, stratified by gender, age, and cancer types. To achieve allocation concealment, the computer-based data management system (REDCap: Research Electronic Data Capture) assigns participants to 1 of 2 groups after the study enrollment procedures. Participants are informed of their group assignment (Group A or B) after the baseline assessment (see Table 1). Participants complete the weekly symptom checklist via Epic MyChart, a patient portal, during the intervention phase (Group A: weeks 1-16 or Group B: weeks 9-16). They are shown how to install the MyChart app on their smartphone and to access it on their personal computer or tablet. If they do not have a MyChart account, study personnel assist them in establishing one. The study team provides a smartphone during the study period if participants do not have one or are not willing to use their own device for the study.

**Table 1:** Study parameters that are completed at baseline, week 8, and week 16.

Study measure	Baseline (week 0)	Weeks 1-8	Week 8	Weeks 9-16	Week 16
Symptom severity item (1 item)^a^	P^b^	—^c^	—	—	—
Sociodemographic form	P	—	—	—	—
SyMon-SAYS^d^ symptom checklist^e^	—	C^f^	—	C	—
Symptom management barriers questionnaire	P	—	P	—	P
PROMIS^g^ HRQOL^h^	C (P)	—	C (P)	—	C (P)
NIH toolbox self-efficacy^i^	C	—	C	—	C
Health-LiTT^j^	P	—	—	—	—
Program evaluation (EXIT Survey)	—	—	—	—	P, M^k^, C

^a^How much are you bothered by symptoms of your condition, or side effects of treatment ? (Not at all; A little; A lot)

^b^P: parent.

^c^Not available.

^d^SyMon-SAYS: symptom monitoring and systematic assessment and reporting system in young survivors.

^e^Symptoms included in this scale are tiredness, pain, insomnia, lack of appetite, worry, nausea, sadness, itch, and headache.

^f^C: Child; R: Research Assistant (ie, chart review).

^g^PROMIS: patient-reported outcome measurement information system.

^h^Health-related quality of life (HRQoL) Domains are: Depression, Anxiety, Anger, Fatigue, Mobility, Upper Extremity Function, Peer Relationships.

^i^NIH: national institutes of health.

^j^Health-LiTT: health literacy assessment using talking touchscreen technology short-form version, which can and will be administered without talking touchscreen technology in this study.

^k^M: MD and RN.

### Study Implementation

#### Overview

All participants (Group A and B as shown in Figure 2) experience the intervention phase during the study—Group A during weeks 1-8 and weeks 9-16 and Group B during weeks 9-16 (see Figure 2). Hereafter, intervention phase participants will be referred to as “IP.” After enrollment, study personnel demonstrate how to complete baseline assessments using an iPad. Intervention participant (IP) participants are trained on accessing SyMon-SAYS using the Epic MyChart patient portal. IP participants are provided with a card containing their unique ID code and brief instructions for using the system. The card also contains the study personnel’s name and telephone number for any questions that might arise. IP participants are asked to choose a standard day of the week to access the system and complete their weekly symptom assessment. Parents of IP participants are informed that the study team will send a reminder to them 1 day prior to the chosen day regarding the upcoming assessment. IP participants are given a window of 1 business day before and 2 business days after their preferred day to access the system. IP participants complete the SyMon-SAYS symptom checklist (symptom items are available in Multimedia Appendix 1) every week for 8 weeks through Epic MyChart via mobile app, computer, or tablet. If IP participants do not access the system by midnight of the preferred day, study personnel contact the parent the following day (preferred day +1 business day) and remind them to complete the assessment or prompt their child to do so. If the participant does not access the system by midnight of the day following their preferred day, study personnel again attempt to contact the parent by phone (preferred day +2 business days) to remind them or prompt their child to access the system. Study personnel ascertains any issues that might be related to noncompliance (eg, hospitalization) during reminder calls. At weeks 8 and 16, all participants complete the measures listed in Table 1.

#### Symptom Clinical Intervention Triggers (Intervention Phase Only)

Children’s symptom scores will be automatically monitored and reported to oncology care providers. When a child’s symptom score exceeds the preset severity threshold (ie, scores 3 or higher), the system will generate an email alert through Epic messaging to the study team with the child’s study ID. Upon receipt of an alert, the study coordinator will access the report in Epic and notify the child’s treating team to determine whether a call is needed. If needed, the nurse will contact the parent within 1 business day to ascertain their child’s current status (see Figure 3 for study schema). The nurse will document actions taken in Epic.

#### Symptom Monitoring Summary Report (Intervention Phase Only)

After children and parents complete the week 8 and week 16 assessments, study personnel print out symptom reports (Group A: week 8 and week 16; Group B: week 16 only) in clinic and deliver them to parents of IP participants and treating provider prior to their appointment (either week 8 or week 16). Parents are also encouraged to review the web-based version of the report by logging into MyChart prior to clinical visits. Clinicians in the child’s care team are able to review this report in children’s medical records, where it is stored along with other usual clinical data. These reports reflect children’s cumulative symptom scores between baseline, week 8, and week 16 (Group A in Figure 2) or between week 8 to week 16 (Group B). Parents are encouraged to discuss the symptom reports with their child’s treating clinicians.

Additionally, the study coordinator will inform children or parents during enrollment about the symptom management resources website that is linked directly to the symptom report in MyChart. This resource site was created by clinicians at Lurie Children’s Hospital and the study team, which contain guidelines on managing symptoms at home and when to contact clinicians in a timely manner.

### Study Outcomes

#### SyMon-SAYS Weekly Symptom Assessment

This checklist consists of 9 symptoms: tiredness, pain, insomnia, lack of appetite, worry, nausea, sadness, itch, and headache. These symptoms were selected by clinicians at Lurie Children’s Hospital and referenced to our survey of 25 neuro-oncologists and nurses from October-December 2014 in which they were asked to name the top 3 symptoms to be listed on the report. Each symptom is measured by using 1 item with a 5-point rating scale. The SyMon-SAYS study team selected candidate items from existing instruments. Clinicians of Hematology, Oncology, Neuro-Oncology and Stem Cell Transplant Division, Lurie Children’s Hospital reviewed candidate items and finalized items included in the symptom checklist. Final items were drawn or modified from Pediatric Patient-Reported Outcome Measurement Information System (PROMIS) Fatigue, Depressive Symptoms, Anxiety, and Sleep, PROMIS Pediatric Itch Questionnaire – Child, Pediatric Quality of Life in Neurological Disorders–Pain, Pediatric Patient-Reported Outcomes version of the Common Terminology Criteria for Adverse Events (Ped-PRO-CTCAE), Pediatric Functional Assessment of Anorexia/Cachexia Treatment, and Pediatric Functional Assessment of Cancer Therapy – Brain. Symptom Checklist items can be found in the Multimedia Appendix 1. Each item was measured by using a 5-point rating scale. An alert will be generated by SyMon-SAYS via Epic messaging when patients endorse a rating of 3 or higher.

#### Usability, Acceptability, and Engagement of the SyMon-SAYS Program

Feasibility, acceptability, and engagement will be evaluated using mixed methods across child-parent dyads and clinicians.

For the quantitative methodology, we will calculate the percentage of weekly symptom assessments that children complete, the number of weekly symptom scores that exceed a prior threshold to trigger alerts, as well as clinician documentation to symptom alerts in the EHR. In addition, a subset of participants (parent-child dyads) will be interviewed to obtain their feedback on the symptom report discussions with their clinician and their experience participating in the SyMon-SAYS study at the end of the intervention phase. Children and parents will be interviewed independently. Efforts will be made to balance gender (children and parents), ages (children), clinical variables of children as well as English- and Spanish-speaking participants (all children are English-speaking). Participants whose parents speak Spanish will be interviewed in Spanish.

At the completion of the study, clinicians, including attending physicians, fellows, advanced practice nurses and physician assistants, and registered nurses, will also be interviewed by either individual interviews (via telephone) or email survey to obtain their feedback on the symptom report, the study, and suggestions on how to make it more accessible and child friendly.

We plan to interview up to 30 parent-child dyads and up to 20 clinicians. The telephone interviews will be recorded for transcription and subsequent summary of feedback. Members of the study team will use selective qualitative analysis methods and an iterative coding process to identify common themes and develop coding rules to apply to interviewees’ comments. The comments will then be compiled and summarized in frequency tables denoting the number of times certain responses emerge. Both frequency and the relative importance placed on the theme will be used to evaluate usability, acceptability, and engagement along with quantitative results.

#### Adherence to Intervention

The adherence to the SyMon-SAYS Intervention will be evaluated by using the percentage of dyads completing the assessments at all time-points, excluding those who are off-study or deceased.

### Measures Used for Primary and Secondary Outcomes

#### Modified Symptom Management Barriers Questionnaire

The modified Symptom Management Barriers Questionnaire (SMBQ) is a 23-item tool designed to assess the attitude (perceived barriers) of parents with children diagnosed with cancer toward the assessment and management of their child’s symptoms. The SMBQ comprises items that encompass various recognized barriers to achieving effective symptom management. The original version of the SMBQ [9] items was devised with input from experts in symptom assessment and management and interviews with patients and their spouses. The study team made adjustments to the items to ensure their suitability for parents of children with cancer, such as replacing the pronoun “I” with “my child.”

#### Pediatric PROMIS

Change in HRQoL over time will be analyzed by examining changes in the pediatric PROMIS measures. We will administer computerized adaptive tests (CATs) of the domains in the PROMIS Pediatric Profile [50]: (1) physical function-mobility, (2) physical function-upper extremity, (3) depressive symptoms, (4) anxiety, (5) anger, and (6) relationship with peers. The pediatric PROMIS offer good reliability across 2-3 standard deviations of the experiences of children 8-17 years old.

#### Self-Efficacy: NIH Toolbox Self-Efficacy

The National Institutes of Health (NIH) Toolbox initiative was developed to identify, create, and validate measures in the broad domains of cognitive function, emotional health, motor function, and sensory function [51,52]. Measures of self-efficacy, defined as a person’s belief in their capacity to manage their functioning and have control over meaningful events, were developed within the emotional health domain. This measure is available for children aged 8-12 years, adolescents aged 13-17 years, and adults aged 18 years or older and is available as CAT. These measures demonstrated good psychometric properties; US general population norms are available. Patients and parents will complete age-appropriate CATs.

### Statistical Analysis

#### Analysis for Aim 1: Evaluate Efficacy of SyMon-SAYS After 8 Weeks and its Maintenance Effects at Week 16

The coprimary outcomes of the study will be perceived symptom management barriers, child’s and parent’s self-efficacy, and child’s anxiety and depression. Data obtained from baseline, week 8, and week 16 will be analyzed. The primary analysis of intervention efficacy will focus on differences in changes between Group A and Group B in perceived barriers to managing symptoms, self-efficacy and depression, and anxiety from baseline to week 8. We will fit mixed effects models for repeated measures. Barriers, child and parent self-efficacy, depression, and anxiety will each be examined separately as dependent variables in these models (ie, each with separate models). We also will explore the associations of demographic variables (eg, gender), individual factors (eg, health literacy), and clinical characteristics (eg, intensity of treatment) on primary outcomes. We will control for multiplicity among coprimary outcomes with the Benjamini-Hochberg [53] procedure.

Secondary outcomes are changes in other domains of HRQoL, including fatigue, mobility, and peer relationships, as well as symptom burden. We will use the same approach to changes in patients’ PROMIS Fatigue, PROMIS Mobility, and PROMIS Peer Relationships will take the same approach as that described above for primary outcomes using mixed effects models. Regarding from baseline to week 8 (Group A) or from week 9 to week 16 (Group B) estimated by calculating the area under the curve (AUC) of each symptom score plotted over time for each participant. The AUC will be then divided by the total time to rescale back to the original units. We will calculate a *P* value for differences in AUC curves between arms with a Wilcoxon rank test.

We hypothesize that group A participants will report fewer perceived barriers, less symptom burden, better self-efficacy, and better HRQoL over phase 1 (weeks 1-8) and then either stay the same or continue to improve over phase 2 (weeks 9-16; exploratory end points), when they continue to receive the SyMon-SAYS intervention. Group B participants, on the other hand, are not expected to report significantly different scores from baseline to the end of phase 1 (ie, week 8) but are expected to report significantly improved outcomes at week 16 than the scores at the baseline.

#### Analysis for Aim 2: Evaluate Factors Associated With Aim 1 Efficacy Outcomes

For each of the Aim 1 outcomes, we will code individual patients as having improved or not improved by a clinically important threshold. Then, we will examine whether patient and parent contextual factors (eg, patient demographic variables, parent health literacy, and disease severity) are associated with improvement in each outcome (each modeled separately) using multivariable logistic regression models.

#### Analysis for Aim 3: Evaluate Predictors of Adherence to the SyMon-SAYS Intervention and Preference of SyMon-SAYS Versus Usual Care

We will estimate participants’ adherence to SyMon-SAYS during the intervention phase (weeks 1-8 for Group A and weeks 9-16 for Group B), defined as percentage of dyads completing the assessments at all time-points. Preference for SyMon-SAYS versus usual care will be assessed using relevant items in the patient and parent EXIT evaluation survey. These item responses will be descriptively summarized with frequency distributions. Clinicians’ perceptions of the acceptability and usefulness of SyMon-SAYS (Evaluation Form) will be similarly assessed by summarizing responses to survey items.

We will identify predictors of intervention adherence and preference using ordinal regression models using data from both groups’ intervention phases (weeks 1-8 for group A, weeks 9-16 for group B). Based on number of weekly system assessments completed, patients will be classified as high adherence (completed 7 or 8 weekly assessments), intermediate adherence (completed 3-6 weekly assessments), or low adherence (completed 1 or 2 or fewer weekly assessments). Parent and patient contextual factors to be examined as predictors such as gender, age, race, parent education, and parent health literacy as well as clinical characteristics such as treatment types. Additionally, we will repeat the same analysis using data from week 9- week 16 from group A to explore whether the group membership (ie, high, intermediate, or low adherence) changed between phase 1 (baseline to week 8) and phase 2 (week 9- week 16) as well as predictors associated with the group membership.

### Data Quality and Management

In order to ensure adequate numbers of patients with data for the primary end point analysis, we increased the sample size to account for 20% dropout. Prior to performing analyses, we will evaluate the amount, reasons, and patterns of missing data. We will compare the baseline characteristics of patients with and without complete data. If necessary, multiple imputations of weekly symptom data for secondary end points by chained equations will be used to impute missing data.

To be in compliance with NCI’s established publications and data-sharing policy for projects that are funded as part of the Beau Biden Cancer Moonshot Initiative, we will create a data codebook that explains each variable in our databases. We will create deidentified databases in easily-readable formats (eg, tab-delimited) for the express purpose of data-sharing. Underlying primary data for the publications will be made broadly available through our Dataverse site [54]. We will make our publications immediately open access. Publication author eligibility guidelines will be followed. Electronic copies of publications will be deposited in PubMed Central with proper tagging of metadata to ensure web-based discoverability and accessibility within 4 weeks of acceptance by a journal.

### Study Timeline

We plan to complete this study in 60 months, including startup (2 months), report development (3 months), assembling symptom management information from web-based resources (8 months), programming SyMon-SAYS (6 months), pilot testing the system, training study personnel, modifying the system based on pilot feedback (6 months), study implementation (36 months, including 16 weeks follow-up), data analysis and manuscript preparation (6 months). Recruitment began in April 2021 and is expected to be completed in Spring or Summer, 2024.

## Results

This study has received approval from the institutional review boards of Northwestern University (STU00210598) and Ann & Robert Lurie Children’s Hospital of Chicago (IRB 2019-3018). Recruitment began in April 2021 and is anticipated to be completed by Spring or Summer 2024. Following the completion of data collection and analysis, the results will be submitted for publication in a manuscript describing the updated trial results.

## Discussion

SyMon-SAYS has several unique features that will significantly advance self-management through incorporating patient-reported symptom monitoring with IT. SyMon-SAYS is incorporated into the EHR to minimize clinic workflow disruption to clinicians and facilitate accessibility by parents and patients, thereby promoting adherence in children, parents and clinicians. Integrating PROs into the EHR has shown improved outcomes in adult patients with cancer [55]. Similar efforts in patients with pediatric cancer are still in their infancy with either focused on patients with pediatric cancer who were hospitalized [37] or were not incorporated into electronic medical records [56]. While SyMon-SAYS captures symptoms occurring between clinical visits. SyMon-SAYS produces user-friendly graphic symptom reports of scores displaying change over time that are presented to parents and clinicians. Parents (and clinicians) will be able to track patients’ symptom levels over time, which can enhance their awareness of symptom variability, contribute to increased symptom and illness or disease knowledge, seek medical help in a timely manner, and promote their ability to prepare for communicating with the clinical team [57].

The study has some potential caveats that will need to be considered in interpreting the findings and should be addressed in future research. We considered several randomization strategies, including cluster randomization by physician versus individual-level randomization by patient-parent dyad. Due to the limited number of eligible physicians within a single institution, we deemed it best to randomize by patient despite the known limitations, the greatest of which is contamination. Although exposure to the intervention may influence physicians’ discussion or management of their patients in the waitlist control (group B, weeks 1-8), they will not have access to symptom reports for their patients who have not yet crossed over from control to intervention. Given the potential for interinstitutional variability in functionalities to support routine symptom surveillance, we elected to conduct a single institution randomized phase 2 trial to detect an efficacy signal prior to moving to a pivotal phase 3 trial across multiple institutions if our findings demonstrate that the intervention is efficacious. This future trial would randomize by the clinic. We chose a waitlist control design primarily because it exposes Group B participants to both usual care and the SyMon-SAYS intervention, allowing them to state their preferred approach for symptom management. Instead of a cross-over design, we chose to have group A participants continue intervention during weeks 9-16 as it is not reasonable to discourage them from accessing reports and resources available in the Epic EHR that they are exposed to in weeks 1-8. Additionally, this approach allows us to evaluate the durability of the SyMon-SAYS intervention. This study will provide a better understanding of factors impacting patients’ and parents’ preferences, which can inform the development of individualized interventions to manage symptoms more effectively. We will review the frequency with which participants access web-based information resources. Group differences in frequencies of accessing information resources will not be evaluated during weeks 9-16, as there may be a sustained benefit from weeks 1-8. Thus, the primary comparisons will occur using data collected during the first 8 weeks.

### Conclusions

In conclusion, we believe that this randomized waitlist controlled trial of the SyMon-SAYS system will provide preliminary efficacy estimates, gauge feasibility and acceptability, identify predictors of engagement, and suggest strategies to strengthen adherence and adoption in subsequent trials of digital symptom monitoring and management tools in children with cancer across the disease continuum, from active therapy to long-term follow-up.

## Appendix

Multimedia Appendix 1 Symptom monitoring and systematic assessment and reporting system in young survivors (SyMon-SAYS) weekly symptom checklist.

Multimedia Appendix 2 CONSORT e-HEALTH checklist (V 1.6.1).

## Abbreviations

AUC: area under the curve
CAT: computerized adaptive test
DSMB: Data and Safety Monitoring Board
EHR: electronic health record
HRQoL: health-related quality of life
IP: intervention participant
NIH: National Institutes of Health
PI: principal investigator
Ped-PRO-CTCAE: pediatric patient-reported outcomes version of the common terminology criteria for adverse events
PROMIS: Pediatric Patient-Reported Outcome Measurement Information System
SyMon-SAYS: Symptom Monitoring and Systematic Assessment and Reporting System in Young Survivors

## Data Availability

Once the primary results are published, the underlying primary data for the publications will be made widely accessible through the Dataverse [58].
