# Supplementary material for: Using IT to Improve Outcomes for Children Living With Cancer (SyMon-SAYS): Protocol for a Single-Institution Waitlist Randomized Controlled Trial
Source: JMIR Res Protoc. 2023 Sep 8;12:e50993. doi: 10.2196/50993 (PMC10517385; doi:10.2196/50993)
Supplement: Multimedia Appendix 1 [file resprot_v12i1e50993_app1.docx]

Symptom monitoring and systematic assessment and reporting system in young survivors (SyMon-SAYS) weekly symptom checklist.

| Symptom | Item | Rating scale^a^ | Original instrument |
| --- | --- | --- | --- |
| Tiredness | In the past 7 days, I got tired easily | 1=Never  2=Almost never  3=Sometimes  4=Often  5=Almost always | PROMIS Pediatric Fatigue (PROMIS Pediatric Profile-25)^b^ |
| Sadness | In the past 7 days, I felt sad | 1=Never  2=Almost never  3=Sometimes  4=Often  5=Almost always | PROMIS Pediatric Depressive Symptoms (PROMIS Profile-25)^b^ |
| Itch | In the past 7 days, how bad was your itch on average? | 1=No itch  2=Mild  3=Moderate  4=Severe  5=Very Severe | PROMIS Pediatric Itch Questionnaire-Child (PIQ-C) |
| Pain | In the past 7 days, I had a lot of pain | 1=Never  2=Almost never  3=Sometimes  4=Often  5=Almost always | pediatric Quality of Life in Neurological Disorders (Neuro-QOL)-Pain |
| Worry | In the past 7 days, I felt worried | 1=Never  2=Almost never  3=Sometimes  4=Often  5=Almost always | PROMIS Pediatric Anxiety (PROMIS Profile-25)^b^ |
| Appetite | In the past 7 days, how often did you not want to eat your meals? | 1=Never  2=Almost never  3=Sometimes  4=Often  5=Almost always | Modified from Pediatric PRO-CTCAE^c^ |
| Vomit/Nausea | In the past 7 days, I have been throwing up | 1=Not at all  2=A little bit  3=Somewhat  4=Quite often  5=Very much | Pediatric Functional Assessment of Anorexia/Cachexia Treatment (peds FAACT) |
| Insomnia | In the past 7 days, I had trouble sleeping | 1=Never  2=Almost never  3=Sometimes  4=Almost always  5=Always | PROMIS Sleep Disturbance SF 4a |
| Headache | In the past 7 days, I am bothered by headaches | 1=Not at all  2=A little bit  3=Somewhat  4=Quite often  5=Very much | Pediatric Functional Assessment of Cancer Therapy-Brain (peds FACT-Br) |

^a^ A symptom alert is generated when a score of 3 or higher is endorsed by the child.

^b^ This item is included in the PROMIS Profile-25.

^c^ This item was modified from the National Cancer Institute’s Pediatric Patient-Reported Outcomes version of the Common Terminology Criteria for Adverse Events (PRO-CTCAE) [57]. [For more information visit: https://healthcaredelivery.cancer.gov/pro-ctcae/]
