# Supplementary material for: Using IT to Improve Outcomes for Children Living With Cancer (SyMon-SAYS): Protocol for a Single-Institution Waitlist Randomized Controlled Trial
Source: JMIR Res Protoc. 2023 Sep 8;12:e50993. doi: 10.2196/50993 (PMC10517385; doi:10.2196/50993)
Supplement: Multimedia Appendix 2 [file resprot_v12i1e50993_app2.pdf]

CONSORT-EHEALTH (V 1.6.1) - Submission/Publication Form

The CONSORT-EHEALTH checklist is intended for authors of randomized trials evaluating web-based and Internet-based applications/interventions, including mobile interventions, electronic games (incl multiplayer games), social media, certain telehealth applications, and other interactive and/or networked electronic applications. Some of the items (e.g. all subitems under item 5 - description of the intervention) may also be applicable for other study designs.

The goal of the CONSORT EHEALTH checklist and guideline is to be
a) a guide for reporting for authors of RCTs,
b) to form a basis for appraisal of an ehealth trial (in terms of validity)

CONSORT-EHEALTH items/subitems are MANDATORY reporting items for studies published in the Journal of Medical Internet Research and other journals / scientific societies endorsing the checklist.

Items numbered 1., 2., 3., 4a., 4b etc are original CONSORT or CONSORT-NPT (non-pharmacologic treatment) items.
Items with Roman numerals (i., ii, iii, iv etc.) are CONSORT-EHEALTH extensions/clarifications.

As the CONSORT-EHEALTH checklist is still considered in a formative stage, we would ask that you also RATE ON A SCALE OF 1-5 how important/useful you feel each item is FOR THE PURPOSE OF THE CHECKLIST and reporting guideline (optional).

Mandatory reporting items are marked with a red *.
In the textboxes, either copy & paste the relevant sections from your manuscript into this form - please include any quotes from your manuscript in QUOTATION MARKS,
or answer directly by providing additional information not in the manuscript, or elaborating on why the item was not relevant for this study.

YOUR ANSWERS WILL BE PUBLISHED AS A SUPPLEMENTARY FILE TO YOUR PUBLICATION IN JMIR AND ARE CONSIDERED PART OF YOUR PUBLICATION (IF ACCEPTED).
Please fill in these questions diligently. Information will not be copyedited, so please use proper spelling and grammar, use correct capitalization, and avoid abbreviations.

DO NOT FORGET TO SAVE AS PDF _AND_ CLICK THE SUBMIT BUTTON SO YOUR ANSWERS ARE IN OUR DATABASE !!!

Citation Suggestion (if you append the pdf as Appendix we suggest to cite this paper in the caption):
Eysenbach G, CONSORT-EHEALTH Group

CONSORT-EHEALTH: Improving and Standardizing Evaluation Reports of Web-based and Mobile Health Interventions
J Med Internet Res 2011;13(4):e126
URL: http://www.jmir.org/2011/4/e126/
doi: 10.2196/jmir.1923
PMID: 22209829

sally.e.jensen@gmail.com Switch account

Not shared

Draft saved

* Indicates required question

Your name *

First Last

Jin-Shei Lai

Primary Affiliation (short), City, Country *

University of Toronto, Toronto, Canada

Northwestern University, Chicago, IL

Your e-mail address *

abc@gmail.com

js-lai@northwestern.edu

Title of your manuscript *

Provide the (draft) title of your manuscript.

Study Protocol fr the Using Information Technology to Improve Outcomes for Children Living with Cancer (SyMon-SAYS): A single institution waitlist control randomized trial

Name of your App/Software/Intervention *

If there is a short and a long/alternate name, write the short name first and add the long name in brackets.

SyMon-SAYS

Evaluated Version (if any)

e.g. "V1", "Release 2017-03-01", "Version 2.0.27913"

Your answer

Language(s) *

What language is the intervention/app in? If multiple languages are available, separate by comma (e.g. "English, French")

English

URL of your Intervention Website or App

e.g. a direct link to the mobile app on app in appstore (itunes, Google Play), or URL of the website. If the intervention is a DVD or hardware, you can also link to an Amazon page.

Your answer

URL of an image/screenshot (optional)

Your answer

Accessibility *

Can an enduser access the intervention presently?

- ○ access is free and open
- ○ access only for special usergroups, not open
- ○ access is open to everyone, but requires payment/subscription/in-app purchases
- ○ app/intervention no longer accessible
- ◉ Other: Intervention is incorporated into a single healthcare system electronic

Primary Medical Indication/Disease/Condition *

e.g. "Stress", "Diabetes", or define the target group in brackets after the condition, e.g. "Autism (Parents of children with)", "Alzheimers (Informal Caregivers of)"

Children and Adolescents with Cancer

Primary Outcomes measured in trial *

comma-separated list of primary outcomes reported in the trial

Perceived barriers to symptom management, [illegible]

Secondary/other outcomes

Are there any other outcomes the intervention is expected to affect?

Your answer

Recommended "Dose" *

What do the instructions for users say on how often the app should be used?

- ○ Approximately Daily
- ◉ Approximately Weekly
- ○ Approximately Monthly
- ○ Approximately Yearly
- ○ "as needed"
- ○ Other:

Approx. Percentage of Users (starters) still using the app as recommended after 3 months *

- (selected) unknown / not evaluated
- 0-10%
- 11-20%
- 21-30%
- 31-40%
- 41-50%
- 51-60%
- 61-70%
- 71%-80%
- 81-90%
- 91-100%
- Other:

Overall, was the app/intervention effective? *

- yes: all primary outcomes were significantly better in intervention group vs control
- partly: SOME primary outcomes were significantly better in intervention group vs control
- no statistically significant difference between control and intervention
- potentially harmful: control was significantly better than intervention in one or more outcomes
- inconclusive: more research is needed
- (selected) Other: To be determined. Data collection is ongoing

Article Preparation Status/Stage *

At which stage in your article preparation are you currently (at the time you fill in this form)

- ○ not submitted yet - in early draft status
- ◉ not submitted yet - in late draft status, just before submission
- ○ submitted to a journal but not reviewed yet
- ○ submitted to a journal and after receiving initial reviewer comments
- ○ submitted to a journal and accepted, but not published yet
- ○ published
- ○ Other:

Journal *

If you already know where you will submit this paper (or if it is already submitted), please provide the journal name (if it is not JMIR, provide the journal name under "other")

- ○ not submitted yet / unclear where I will submit this
- ○ Journal of Medical Internet Research (JMIR)
- ○ JMIR mHealth and UHealth
- ○ JMIR Serious Games
- ○ JMIR Mental Health
- ○ JMIR Public Health
- ○ JMIR Formative Research
- ○ Other JMIR sister journal
- ◉ Other: JMIR Research Protocols

Is this a full powered effectiveness trial or a pilot/feasibility trial? *

- ○ Pilot/feasibility
- ◉ Fully powered

Manuscript tracking number *

If this is a JMIR submission, please provide the manuscript tracking number under "other" (The ms tracking number can be found in the submission acknowledgement email, or when you login as author in JMIR. If the paper is already published in JMIR, then the ms tracking number is the four-digit number at the end of the DOI, to be found at the bottom of each published article in JMIR)

- ◉ no ms number (yet) / not (yet) submitted to / published in JMIR
- ○ Other:

TITLE AND ABSTRACT

1a) TITLE: Identification as a randomized trial in the title

1a) Does your paper address CONSORT item 1a? *

I.e does the title contain the phrase "Randomized Controlled Trial"? (if not, explain the reason under "other")

- ◉ yes
- ○ Other:

1a-i) Identify the mode of delivery in the title

Identify the mode of delivery. Preferably use “web-based” and/or “mobile” and/or “electronic game” in the title. Avoid ambiguous terms like “online”, “virtual”, “interactive”. Use “Internet-based” only if Intervention includes non-web-based Internet components (e.g. email), use “computer-based” or “electronic” only if offline products are used. Use “virtual” only in the context of “virtual reality” (3-D worlds). Use “online” only in the context of “online support groups”. Complement or substitute product names with broader terms for the class of products (such as “mobile” or “smart phone” instead of “iphone”), especially if the application runs on different platforms.

subitem not at all important

1 ○

2 ○

3 ○

4 ○

5 ◉

essential

Clear selection

Does your paper address subitem 1a-i? *

Copy and paste relevant sections from manuscript title (include quotes in quotation marks "like this" to indicate direct quotes from your manuscript), or elaborate on this item by providing additional information not in the ms, or briefly explain why the item is not applicable/relevant for your study

"Study Protocol for the Using Information Technology to Improve Outcomes for Children Living with Cancer (SyMon-SAYS)"

1a-ii) Non-web-based components or important co-interventions in title

Mention non-web-based components or important co-interventions in title, if any (e.g., “with telephone support”).

subitem not at all important

1 ○

2 ○

3 ◉

4 ○

5 ○

essential

Clear selection

Does your paper address subitem 1a-ii?

Copy and paste relevant sections from manuscript title (include quotes in quotation marks "like this" to indicate direct quotes from your manuscript), or elaborate on this item by providing additional information not in the ms, or briefly explain why the item is not applicable/relevant for your study

"Using Information Technology to Improve Outcomes for Children Living with Cancer (SyMon-SAYS)"
This is an information technology system which is integrated into the electronic health record through which participants provide data using EPIC MyChart, which can be used from any device with internet connectivity.

1a-iii) Primary condition or target group in the title

Mention primary condition or target group in the title, if any (e.g., “for children with Type I Diabetes”) Example: A Web-based and Mobile Intervention with Telephone Support for Children with Type I Diabetes: Randomized Controlled Trial

subitem not at all important

1 ○

2 ○

3 ○

4 ○

5 ◉

essential

Clear selection

Does your paper address subitem 1a-iii? *

Copy and paste relevant sections from manuscript title (include quotes in quotation marks "like this" to indicate direct quotes from your manuscript), or elaborate on this item by providing additional information not in the ms, or briefly explain why the item is not applicable/relevant for your study

"for Children Living with Cancer"

1b) ABSTRACT: Structured summary of trial design, methods, results, and conclusions

NPT extension: Description of experimental treatment, comparator, care providers, centers, and blinding status.

1b-i) Key features/functionalities/components of the intervention and comparator in the METHODS section of the ABSTRACT

Mention key features/functionalities/components of the intervention and comparator in the abstract. If possible, also mention theories and principles used for designing the site. Keep in mind the needs of systematic reviewers and indexers by including important synonyms. (Note: Only report in the abstract what the main paper is reporting. If this information is missing from the main body of text, consider adding it)

subitem not at all important

- 1 ○
- 2 ○
- 3 ○
- 4 ○
- 5 ◉

essential

Clear selection

Does your paper address subitem 1b-i? *

Copy and paste relevant sections from the manuscript abstract (include quotes in quotation marks "like this" to indicate direct quotes from your manuscript), or elaborate on this item by providing additional information not in the ms, or briefly explain why the item is not applicable/relevant for your study

"The SyMon-SAYS system is integrated into the EHR to streamline the presentation of symptom scores and delivery of alerts for severe symptons to clinicians using EHR (Epic) messaging functionalities.Children (ages 8 to 17) complete the weekly symptom assessment and review of the symptom report by logging into the patient portal (Epic MyChart). "

1b-ii) Level of human involvement in the METHODS section of the ABSTRACT

Clarify the level of human involvement in the abstract, e.g., use phrases like “fully automated” vs. “therapist/nurse/care provider/physician-assisted” (mention number and expertise of providers involved, if any). (Note: Only report in the abstract what the main paper is reporting. If this information is missing from the main body of text, consider adding it)

subitem not at all important

- 1 ○
- 2 ○
- 3 ○
- 4 ◉
- 5 ○

essential

Clear selection

Does your paper address subitem 1b-ii?

Copy and paste relevant sections from the manuscript abstract (include quotes in quotation marks "like this" to indicate direct quotes from your manuscript), or elaborate on this item by providing additional information not in the ms, or briefly explain why the item is not applicable/relevant for your study

"The SyMon-SAYS system is integrated into the EHR to streamline the presentation of symptom scores and delivery of alerts for severe symptons to clinicians using EHR (Epic) messaging functionalities.Children (ages 8 to 17) complete the weekly symptom assessment and review of the symptom report by logging into the patient portal (Epic MyChart). "

1b-iii) Open vs. closed, web-based (self-assessment) vs. face-to-face assessments in the METHODS section of the ABSTRACT

Mention how participants were recruited (online vs. offline), e.g., from an open access website or from a clinic or a closed online user group (closed usergroup trial), and clarify if this was a purely web-based trial, or there were face-to-face components (as part of the intervention or for assessment). Clearly say if outcomes were self-assessed through questionnaires (as common in web-based trials). Note: In traditional offline trials, an open trial (open-label trial) is a type of clinical trial in which both the researchers and participants know which treatment is being administered. To avoid confusion, use “blinded” or “unblinded” to indicated the level of blinding instead of “open”, as “open” in web-based trials usually refers to “open access” (i.e. participants can self-enrol). (Note: Only report in the abstract what the main paper is reporting. If this information is missing from the main body of text, consider adding it)

subitem not at all important

1 ○

2 ○

3 ○

4 ○

5 ◉

essential

Clear selection

Does your paper address subitem 1b-iii?

Copy and paste relevant sections from the manuscript abstract (include quotes in quotation marks "like this" to indicate direct quotes from your manuscript), or elaborate on this item by providing additional information not in the ms, or briefly explain why the item is not applicable/relevant for your study

"Children (ages 8 to 17) complete the weekly symptom assessment and review of the symptom report by logging into the patient portal (Epic MyChart). "

1b-iv) RESULTS section in abstract must contain use data

Report number of participants enrolled/assessed in each group, the use/uptake of the intervention (e.g., attrition/adherence metrics, use over time, number of logins etc.), in addition to primary/secondary outcomes. (Note: Only report in the abstract what the main paper is reporting. If this information is missing from the main body of text, consider adding it)

subitem not at all important

- 1 (selected)
- 2
- 3
- 4
- 5

essential

Clear selection

Does your paper address subitem 1b-iv?

Copy and paste relevant sections from the manuscript abstract (include quotes in quotation marks "like this" to indicate direct quotes from your manuscript), or elaborate on this item by providing additional information not in the ms, or briefly explain why the item is not applicable/relevant for your study

No, because data collection is ongoing and the purpose of the paper is to describe the protocol, not the final results of the intervention.

1b-v) CONCLUSIONS/DISCUSSION in abstract for negative trials

Conclusions/Discussions in abstract for negative trials: Discuss the primary outcome - if the trial is negative (primary outcome not changed), and the intervention was not used, discuss whether negative results are attributable to lack of uptake and discuss reasons. (Note: Only report in the abstract what the main paper is reporting. If this information is missing from the main body of text, consider adding it)

subitem not at all important

1 ○

2 ○

3 ○

4 ○

5 ○

essential

Does your paper address subitem 1b-v?

Copy and paste relevant sections from the manuscript abstract (include quotes in quotation marks "like this" to indicate direct quotes from your manuscript), or elaborate on this item by providing additional information not in the ms, or briefly explain why the item is not applicable/relevant for your study

No, because data collection is ongoing and the purpose of the paper is to describe the protocol, not the final results of the intervention.

INTRODUCTION

2a) In INTRODUCTION: Scientific background and explanation of rationale

2a-i) Problem and the type of system/solution

Describe the problem and the type of system/solution that is object of the study: intended as stand-alone intervention vs. incorporated in broader health care program? Intended for a particular patient population? Goals of the intervention, e.g., being more cost-effective to other interventions, replace or complement other solutions? (Note: Details about the intervention are provided in “Methods” under 5)

subitem not at all important

1 ○

2 ○

3 ○

4 ○

5 ◉

essential

Clear selection

Does your paper address subitem 2a-i? *

Copy and paste relevant sections from the manuscript (include quotes in quotation marks "like this" to indicate direct quotes from your manuscript), or elaborate on this item by providing additional information not in the ms, or briefly explain why the item is not applicable/relevant for your study

"Factors contributing to inadequate symptom management exist at the patient, healthcare provider and system levels. Healthcare system barriers include the structure and organization of care, reimbursement, logistics, and resources.[6, 7] Healthcare provider barriers include limited time available during a patient encounter,[8] willingness to elicit information from patients,[6, 9] and challenges in obtaining systematic symptom assessment.[10] For very young patients, there is the added complexity regarding uncertainty about the accuracy of self-report. Patient-level barriers include: failure to report symptoms to clinicians;[11] desire to be a “good patient” to avoid conflict,[12-14] concern over side effects of prescribed medications,[12, 15] and a perception that during cancer treatment symptoms are inevitable or untreatable.[4] A patient-centered approach to healthcare can minimize these barriers. Patient-related factors that facilitate self-management include: proactively seeking information, making suggestions, and sharing their opinion about their self-management regimen with providers.[16]

Improvements in the quality of communication between patients and healthcare providers can favorably affect symptom management, including improved information recall,[17] satisfaction,[18] overall HRQoL,[19, 20] and adherence to practitioners' recommendations.[21] According to the IOM consensus report Cancer Care for the Whole Patient: Meeting Psychosocial Health Needs,[22] inadequate communication and lack of patient involvement are particularly worrisome, as effective patient-clinician communication is linked to positive health outcomes. For patients and families, information is crucial to promote a sense of control, decrease emotional distress, support effective self-management, and eliminate disruptions of daily activities.[23, 24] Research on adult oncology patients showed the average patient asks five or fewer questions during a 15-minute doctor's office visit, with a high proportion asking no questions, suggesting that patients generally are not taking an active role in their care.[25] Previous literature suggests the clinician-patient relationship can be strengthened by the simple addition of a “prompt sheet,” encouraging patients to ask questions about treatment and prognosis; oncologists' efforts to address the issues raised by the use of prompts promoted patient confidence to ask about prognosis, alleviated patient anxiety and reduced clinic visit length.[26] Yet most literature examining interventions to strengthen patient-clinician communication about problematic issues has focused on adult patients and there has been limited research on children with cancer. Patients' developmental ages are critical. Children's less developed verbal skills, along with parents' and clinicians' possibly conflicting communication styles and attitudes toward children, might prevent children from adequately communicating symptoms to providers.[27] Consequently, children are less likely than adults to discuss their symptoms with healthcare providers.[28] "

"In this trial, we are testing the efficacy of 8 weeks of SyMon-SAYS, an EHR-based system for routine symptom surveillance with self-management support to children and

2a-ii) Scientific background, rationale: What is known about the (type of) system

Scientific background, rationale: What is known about the (type of) system that is the object of the study (be sure to discuss the use of similar systems for other conditions/diagnoses, if appropiate), motivation for the study, i.e. what are the reasons for and what is the context for this specific study, from which stakeholder viewpoint is the study performed, potential impact of findings [2]. Briefly justify the choice of the comparator.

subitem not at all important

- 1 ○
- 2 ○
- 3 ○
- 4 ◉
- 5 ○

essential

Clear selection

Does your paper address subitem 2a-ii? *

Copy and paste relevant sections from the manuscript (include quotes in quotation marks "like this" to indicate direct quotes from your manuscript), or elaborate on this item by providing additional information not in the ms, or briefly explain why the item is not applicable/relevant for your study

"Healthcare delivery has shifted from inpatient to outpatient or home settings, leading investigators to focus on integrating technology into symptom monitoring and self-management support. The literature suggests that web based symptom management programs in pediatric cancer patients[29] are feasible, may improve care, enhance patient and provider satisfaction, and lessen symptom burden, and generally to not increase provider time and effort. Tsimicalis et al[30] reviewed studies incorporating technology in pediatric oncology settings, identifying several benefits including surmounting logistical difficulties of distance, time, costs, and transport[31-34]; improving access to experienced and specialized health care professionals in rural and remote areas;[35] offering support after hours; and avoiding unplanned hospital visits,[36] The scope of research exploring electronic symptom surveillance systems in pediatric oncology has been constrained, primarily concentrating on palliative care[29] and hospitalized children.[37] Both do not capture symptoms occurred between clinical visits. This study fills a gap in knowledge about the feasibility, acceptability, and effects on self-efficacy and symptom burden of measurement-based symptom care delivered via the EHR and explores implementation challenges and strategies that will need to be addressed to refine this intervention for optimal efficacy and adoption. The aim of this manuscript is to outline the study protocol (version 4; 02/17/2023) for this waitlist

2b) In INTRODUCTION: Specific objectives or hypotheses

Does your paper address CONSORT subitem 2b? *

Copy and paste relevant sections from the manuscript (include quotes in quotation marks "like this" to indicate direct quotes from your manuscript), or elaborate on this item by providing additional information not in the ms, or briefly explain why the item is not applicable/relevant for your study

"In this single institution modified waitlist control randomized trial, 200 children (ages 8-17) with cancer and their parents will participate for 16 weeks (Group A: 16-week SyMon-SAYS intervention; Group B: 8-week usual care and then 8-week SyMon-SAYS intervention). Our study is addressing the following specific aims:

• Aim 1: Evaluate efficacy of SyMon-SAYS after 8 weeks and its maintenance effects at week-16. We hypothesize that Group A (versus Group B) will report decreased parent-perceived barriers to managing their children's symptoms, decreased child symptom burden, increased child and parent self-efficacy, and ultimately increased child HRQoL at week-8. We expect that these differences between Groups A and B will be narrowed at week-16 when Group B completes the 8-week SyMon-SAYS intervention.

• Aim 2: Evaluate factors associated with Aim 1 efficacy outcomes, including but not limited to 1) contextual factors; 2) adherence to the SyMon-SAYS intervention, and 3) symptom communication between clinicians and children/families.

• Aim 3: Evaluate predictors of adherence to the SyMon-SAYS intervention and preference of SyMon-SAYS versus usual care. We will identify predictors of adherence and model their association by using ordinal regressions. Preference of SyMon-SAYS versus usual care will be evaluated using responses from SyMon-SAYS program evaluation. This understanding will help to develop individualized symptom management approaches for better quality and outcomes of care in pediatric oncology ambulatory settings."

METHODS

3a) Description of trial design (such as parallel, factorial) including allocation ratio

Does your paper address CONSORT subitem 3a? *

Copy and paste relevant sections from the manuscript (include quotes in quotation marks "like this" to indicate direct quotes from your manuscript), or elaborate on this item by providing additional information not in the ms, or briefly explain why the item is not applicable/relevant for your study

"The study design is shown in Figure 2. To increase utility and compliance of clinicians, children and parents, the SyMon-SAYS system was built within the Ann and Robert H. Lurie Children’s Hospital of Chicago (Lurie) EHR (Epic). Participants (parents and children) complete assessments in-clinic using an iPad with internet access at baseline, week-8 and week-16. They are randomly assigned to Group A (weeks 1-16: SyMon-SAYS intervention) or Group B (weeks 1-8: usual care; weeks 9-16: SyMon-SAYS intervention) after the baseline assessment. During the intervention weeks (either weeks 1-16 or weeks 9-16), participants complete a weekly symptom assessment wherever they have internet access using their MyChart patient portal, accessible via smartphone, tablet or computer. "

3b) Important changes to methods after trial commencement (such as eligibility criteria), with reasons

Does your paper address CONSORT subitem 3b? *

Copy and paste relevant sections from the manuscript (include quotes in quotation marks "like this" to indicate direct quotes from your manuscript), or elaborate on this item by providing additional information not in the ms, or briefly explain why the item is not applicable/relevant for your study

After study commencement, the eligibility criteria for parents was expanded to include Spanish-speaking parents to expand the scope of eligible patient/parent dyads.

"A parent of eligible children (father, mother or legal guardians) is eligible for the study if s/he demonstrates sufficient fluency in English or Spanish to understand and provide informed consent (either in clinic or via remote recruitment), agrees to complete assessments at all time-points, and has sufficient cognitive and motor abilities to complete surveys via an electronic device (e.g., smartphone, iPAD, etc) or computer. Only one parent per child participates in the study."

3b-i) Bug fixes, Downtimes, Content Changes

Bug fixes, Downtimes, Content Changes: ehealth systems are often dynamic systems. A description of changes to methods therefore also includes important changes made on the intervention or comparator during the trial (e.g., major bug fixes or changes in the functionality or content) (5-iii) and other “unexpected events” that may have influenced study design such as staff changes, system failures/downtimes, etc. [2].

subitem not at all important

- 1 (selected)
- 2
- 3
- 4
- 5

essential

Clear selection

Does your paper address subitem 3b-i?

Copy and paste relevant sections from the manuscript (include quotes in quotation marks "like this" to indicate direct quotes from your manuscript), or elaborate on this item by providing additional information not in the ms, or briefly explain why the item is not applicable/relevant for your study

No bugs or downtimes have been encountered in the intervention system.

4a) Eligibility criteria for participants

Does your paper address CONSORT subitem 4a? *

Copy and paste relevant sections from the manuscript (include quotes in quotation marks "like this" to indicate direct quotes from your manuscript), or elaborate on this item by providing additional information not in the ms, or briefly explain why the item is not applicable/relevant for your study

•" Patients. Eligible participants are those with a diagnosis of a malignancy (including children with primary brain tumors), who are currently receiving cancer-directed therapy or are within 6 months of completing cancer-directed therapy, and who are between 8 and 17 years old at the time of enrollment, English-speaking, have sufficient cognitive and motor abilities to complete surveys via an electronic device (e.g., smartphone, iPAD, etc) or computer, and able and willing to sign assent forms (for those between 12-17 years of age). No formal cognitive testing is conducted; eligibility based on cognitive status is determined by the judgment of their clinicians. Utilization of any assistive device to complete the assessment is acceptable and will be documented.

• Parents. A parent of eligible children (father, mother or legal guardians) is eligible for the study if s/he demonstrates sufficient fluency in English or Spanish to understand and provide informed consent (either in clinic or via remote recruitment), agrees to complete assessments at all time-points, and has sufficient cognitive and motor abilities to complete surveys via an electronic device (e.g., smartphone, iPAD, etc) or computer. Only one parent per child participates in the study.

• Clinicians. Eligible clinicians are oncology attending physicians, fellows, nurse practitioners, and nurses who treat participating children at Ann & Robert Lurie Children's Hospital."

4a-i) Computer / Internet literacy

Computer / Internet literacy is often an implicit “de facto” eligibility criterion - this should be explicitly clarified.

subitem not at all important

1 ○

2 ○

3 ◉

4 ○

5 ○

essential

Clear selection

Does your paper address subitem 4a-i?

Copy and paste relevant sections from the manuscript (include quotes in quotation marks "like this" to indicate direct quotes from your manuscript), or elaborate on this item by providing additional information not in the ms, or briefly explain why the item is not applicable/relevant for your study

Patients "have sufficient cognitive and motor abilities to complete surveys via an electronic device (e.g., smartphone, iPAD, etc) or computer,"

Parents "has sufficient cognitive and motor abilities to complete surveys via an electronic device (e.g., smartphone, iPAD, etc) or computer. "

"After enrollment, study personnel demonstrate how to complete baseline assessments using an iPad. IP participants are trained on accessing SyMon-SAYS using the Epic MyChart patient portal. "

4a-ii) Open vs. closed, web-based vs. face-to-face assessments:

Open vs. closed, web-based vs. face-to-face assessments: Mention how participants were recruited (online vs. offline), e.g., from an open access website or from a clinic, and clarify if this was a purely web-based trial, or there were face-to-face components (as part of the intervention or for assessment), i.e., to what degree got the study team to know the participant. In online-only trials, clarify if participants were quasi-anonymous and whether having multiple identities was possible or whether technical or logistical measures (e.g., cookies, email confirmation, phone calls) were used to detect/prevent these.

subitem not at all important

- 1 ○
- 2 ○
- 3 ○
- 4 ◉
- 5 ○

essential

Clear selection

Does your paper address subitem 4a-ii? *

Copy and paste relevant sections from the manuscript (include quotes in quotation marks "like this" to indicate direct quotes from your manuscript), or elaborate on this item by providing additional information not in the ms, or briefly explain why the item is not applicable/relevant for your study

Patient participants were recruited in-person in-clinic by study personnel following determination of eligiblity in conjuction with treating clinicians. "We are recruiting 200 children with cancer from hematology, oncology and brain tumor clinics at Ann & Robert Lurie Children's Hospital, Chicago "

Clinicians were enrolled as participants in the study in person in-clinic by study personnel. "Oncology clinical providers who treat participants are also enrolled in the study and provide informed consent as research participants per IRB's requirements at Lurie Children's Hospital."

"After enrollment, study personnel demonstrate how to complete baseline assessments using an iPad. IP participants are trained on accessing SyMon-SAYS using the Epic MyChart patient portal."

"Parents of IP participants are informed that the study team will send a reminder to them one day prior to the chosen day regarding the upcoming assessment. "

Although symptom assessments are completed via Epic MyChart, study personnel communicate with participants to provide reminders to complete assessments and provide participants with their longitudinal symptom reports in-clinic.

4a-iii) Information giving during recruitment

Information given during recruitment. Specify how participants were briefed for recruitment and in the informed consent procedures (e.g., publish the informed consent documentation as appendix, see also item X26), as this information may have an effect on user self-selection, user expectation and may also bias results.

subitem not at all important

1 ○

2 ○

3 ○

4 ○

5 ◉

essential

Clear selection

Does your paper address subitem 4a-iii?

Copy and paste relevant sections from the manuscript (include quotes in quotation marks "like this" to indicate direct quotes from your manuscript), or elaborate on this item by providing additional information not in the ms, or briefly explain why the item is not applicable/relevant for your study

"Study personnel explain the aims of the study, what is involved in study participation, and the potential risks and benefits of the study to the potential participants (children, their parents, and providers) so that they can make an informed decision as to whether they wish to participate. This explanation is done in plain, non-exculpatory language that is easy for parents, patients, and providers to understand. Additionally, all participants who are approached for participation in this study are informed that participation in this study is voluntary, and that a decision to not participate will not jeopardize or affect their medical care or their employment status (for clinical providers) in any way. They are informed that they are also free to discontinue participation at any time during the study with no adverse impact to their treatment, overall care or employment status. Clinical provider participants are also be informed that their decision to participate or discontinue participation will not impact their employment status, and the data they provide during the course of their participation will be used for the purpose of this research study only and will not be made available to the institution or any managing or supervisory personnel (e.g., for their merit reviews).

Risks and/or Ethical Issues
There are no known social or legal risks to subjects who participate in this study. Our experience with data collection using similar questions has indicated that few (less than 1% of over 15,000 participants over more than 20 years) are distressed by the content of the questions posed to them. Participants are advised that if they become upset by the questions, they will have the option to talk with a trained mental health professional. The PI will be notified of all requests for consultation with a mental health professional. There is a slight possibility that enhanced monitoring of symptoms could lead to an increase in anxiety. Yet our feasibility study showed that SyMon-SAYS significantly decreased children's anxiety level at week-4 ($p=0.046$; unpublished data). Furthermore, in the current study, children's emotional distress (worry and sadness) are included in the weekly assessments and are monitored closely. As with all symptoms being monitored, an alert is generated when children's reports of worry and/or sadness meet or exceed the pre-established threshold. Clinicians are notified within 24 hours and take appropriate actions to respond to these symptom experiences. In addition, in consultation with the site clinical team, the study team reserves the right to withdraw any participant for whom there is concern that the intervention is having an adverse effect.

Measures to Protect Privacy and Confidentiality of Participants
Study personnel enroll patients and their parents and assign each patient and parent participant their unique identification (ID) code, which is used for identifying and tracking participants throughout the study. Parent codes are linked to corresponding patient (child/adolescent) codes. Clinician participants are recruited by Drs. Lai and Lenzen and are also be assigned a unique ID code. All data collected from all participants is linked with this unique identifier and not the participants. Information of linking IDs to participants is saved as a separate password protected file that only the PI and the study coordinator can access."

4b) Settings and locations where the data were collected

Does your paper address CONSORT subitem 4b? *

Copy and paste relevant sections from the manuscript (include quotes in quotation marks "like this" to indicate direct quotes from your manuscript), or elaborate on this item by providing additional information not in the ms, or briefly explain why the item is not applicable/relevant for your study

"This study was approved by Institutional Review Boards of Northwestern University (STU00210598) and Ann & Robert Lurie Children's Hospital of Chicago (IRB 2019-3018). Lurie Children's maintains records of the authorization/reliance agreements. "

"To increase utility and compliance of clinicians, children and parents, the SyMon-SAYS system was built within the Ann and Robert H. Lurie Children's Hospital of Chicago (Lurie) EHR (Epic). "

"We are recruiting 200 children with cancer from hematology, oncology and brain tumor clinics at Ann & Robert Lurie Children's Hospital, Chicago"

4b-i) Report if outcomes were (self-)assessed through online questionnaires

Clearly report if outcomes were (self-)assessed through online questionnaires (as common in web-based trials) or otherwise.

subitem not at all important

1 ○

2 ○

3 ○

4 ○

5 ◉

essential

Clear selection

Does your paper address subitem 4b-i? *

Copy and paste relevant sections from the manuscript (include quotes in quotation marks "like this" to indicate direct quotes from your manuscript), or elaborate on this item by providing additional information not in the ms, or briefly explain why the item is not applicable/relevant for your study

"Participants (parents and children) complete assessments in-clinic using an iPad with internet access at baseline, week-8 and week-16. They are randomly assigned to Group A (weeks 1-16: SyMon-SAYS intervention) or Group B (weeks 1-8: usual care; weeks 9-16: SyMon-SAYS intervention) after the baseline assessment. During the intervention weeks (either weeks 1-16 or weeks 9-16), participants complete a weekly symptom assessment wherever they have internet access using their MyChart patient portal, accessible via smartphone, tablet or computer. "

4b-ii) Report how institutional affiliations are displayed

Report how institutional affiliations are displayed to potential participants [on ehealth media], as affiliations with prestigious hospitals or universities may affect volunteer rates, use, and reactions with regards to an intervention.(Not a required item – describe only if this may bias results)

subitem not at all important

1 ○

2 ○

3 ○

4 ○

5 ○

essential

Does your paper address subitem 4b-ii?

Copy and paste relevant sections from the manuscript (include quotes in quotation marks "like this" to indicate direct quotes from your manuscript), or elaborate on this item by providing additional information not in the ms, or briefly explain why the item is not applicable/relevant for your study

Your answer

5) The interventions for each group with sufficient details to allow replication, including how and when they were actually administered

5-i) Mention names, credential, affiliations of the developers, sponsors, and owners

Mention names, credential, affiliations of the developers, sponsors, and owners [6] (if authors/evaluators are owners or developer of the software, this needs to be declared in a “Conflict of interest” section or mentioned elsewhere in the manuscript).

subitem not at all important

1 ○

2 ○

3 ○

4 ○

5 ○

essential

Does your paper address subitem 5-i?

Copy and paste relevant sections from the manuscript (include quotes in quotation marks "like this" to indicate direct quotes from your manuscript), or elaborate on this item by providing additional information not in the ms, or briefly explain why the item is not applicable/relevant for your study

Your answer

5-ii) Describe the history/development process

Describe the history/development process of the application and previous formative evaluations (e.g., focus groups, usability testing), as these will have an impact on adoption/use rates and help with interpreting results.

subitem not at all important

1 ○

2 ○

3 ○

4 ○

5 ○

essential

Does your paper address subitem 5-ii?

Copy and paste relevant sections from the manuscript (include quotes in quotation marks "like this" to indicate direct quotes from your manuscript), or elaborate on this item by providing additional information not in the ms, or briefly explain why the item is not applicable/relevant for your study

Your answer

5-iii) Revisions and updating

Revisions and updating. Clearly mention the date and/or version number of the application/intervention (and comparator, if applicable) evaluated, or describe whether the intervention underwent major changes during the evaluation process, or whether the development and/or content was “frozen” during the trial. Describe dynamic components such as news feeds or changing content which may have an impact on the replicability of the intervention (for unexpected events see item 3b).

subitem not at all important

1 ○

2 ○

3 ○

4 ○

5 ○

essential

Does your paper address subitem 5-iii?

Copy and paste relevant sections from the manuscript (include quotes in quotation marks "like this" to indicate direct quotes from your manuscript), or elaborate on this item by providing additional information not in the ms, or briefly explain why the item is not applicable/relevant for your study

Your answer

5-iv) Quality assurance methods

Provide information on quality assurance methods to ensure accuracy and quality of information provided [1], if applicable.

subitem not at all important

1 ○

2 ○

3 ○

4 ○

5 ○

essential

Does your paper address subitem 5-iv?

Copy and paste relevant sections from the manuscript (include quotes in quotation marks "like this" to indicate direct quotes from your manuscript), or elaborate on this item by providing additional information not in the ms, or briefly explain why the item is not applicable/relevant for your study

Your answer

5-v) Ensure replicability by publishing the source code, and/or providing screenshots/screen-capture video, and/or providing flowcharts of the algorithms used

Ensure replicability by publishing the source code, and/or providing screenshots/screen-capture video, and/or providing flowcharts of the algorithms used. Replicability (i.e., other researchers should in principle be able to replicate the study) is a hallmark of scientific reporting.

subitem not at all important

1 ○

2 ○

3 ○

4 ○

5 ○

essential

Does your paper address subitem 5-v?

Copy and paste relevant sections from the manuscript (include quotes in quotation marks "like this" to indicate direct quotes from your manuscript), or elaborate on this item by providing additional information not in the ms, or briefly explain why the item is not applicable/relevant for your study

Your answer

5-vi) Digital preservation

Digital preservation: Provide the URL of the application, but as the intervention is likely to change or disappear over the course of the years; also make sure the intervention is archived (Internet Archive, webcitation.org, and/or publishing the source code or screenshots/videos alongside the article). As pages behind login screens cannot be archived, consider creating demo pages which are accessible without login.

subitem not at all important

1 ○

2 ○

3 ○

4 ○

5 ○

essential

Does your paper address subitem 5-vi?

Copy and paste relevant sections from the manuscript (include quotes in quotation marks "like this" to indicate direct quotes from your manuscript), or elaborate on this item by providing additional information not in the ms, or briefly explain why the item is not applicable/relevant for your study

Your answer

5-vii) Access

Access: Describe how participants accessed the application, in what setting/context, if they had to pay (or were paid) or not, whether they had to be a member of specific group. If known, describe how participants obtained “access to the platform and Internet” [1]. To ensure access for editors/reviewers/readers, consider to provide a “backdoor” login account or demo mode for reviewers/readers to explore the application (also important for archiving purposes, see vi).

subitem not at all important

1 ○

2 ○

3 ○

4 ◉

5 ○

essential

Clear selection

Does your paper address subitem 5-vii? *

Copy and paste relevant sections from the manuscript (include quotes in quotation marks "like this" to indicate direct quotes from your manuscript), or elaborate on this item by providing additional information not in the ms, or briefly explain why the item is not applicable/relevant for your study

"To increase utility and compliance of clinicians, children and parents, the SyMon-SAYS system was built within the Ann and Robert H. Lurie Children’s Hospital of Chicago (Lurie) EHR (Epic). Participants (parents and children) complete assessments in-clinic using an iPad with internet access at baseline, week-8 and week-16. They are randomly assigned to Group A (weeks 1-16: SyMon-SAYS intervention) or Group B (weeks 1-8: usual care; weeks 9-16: SyMon-SAYS intervention) after the baseline assessment. During the intervention weeks (either weeks 1-16 or weeks 9-16), participants complete a weekly symptom assessment wherever they have internet access using their MyChart patient portal, accessible via smartphone, tablet or computer. "

5-viii) Mode of delivery, features/functionalities/components of the intervention and comparator, and the theoretical framework

Describe mode of delivery, features/functionalities/components of the intervention and comparator, and the theoretical framework [6] used to design them (instructional strategy [1], behaviour change techniques, persuasive features, etc., see e.g., [7, 8] for terminology). This includes an in-depth description of the content (including where it is coming from and who developed it) [1]," whether [and how] it is tailored to individual circumstances and allows users to track their progress and receive feedback" [6]. This also includes a description of communication delivery channels and – if computer-mediated communication is a component – whether communication was synchronous or asynchronous [6]. It also includes information on presentation strategies [1], including page design principles, average amount of text on pages, presence of hyperlinks to other resources, etc. [1].

subitem not at all important

1 ○

2 ○

3 ○

4 ◉

5 ○

essential

Clear selection

Does your paper address subitem 5-viii? *

Copy and paste relevant sections from the manuscript (include quotes in quotation marks "like this" to indicate direct quotes from your manuscript), or elaborate on this item by providing additional information not in the ms, or briefly explain why the item is not applicable/relevant for your study

"After enrollment, study personnel demonstrate how to complete baseline assessments using an iPad. IP participants are trained on accessing SyMon-SAYS using the Epic MyChart patient portal. IP participants are provided with a card containing their unique ID code and brief instructions for using the system. The card also contains the study personnel's name and telephone number for any questions that might arise. IP participants are asked to choose a standard day of the week to access the system and complete their weekly symptom assessment. Parents of IP participants are informed that the study team will send a reminder to them one day prior to the chosen day regarding the upcoming assessment. IP participants are given a window of one business day before and two business days after their preferred day to access the system. IP participants complete the SyMon-SAYS symptom checklist (symptom items are available in Appendix) every week for 8 weeks through Epic MyChart via mobile app, computer or tablet. If IP participants do not access the system by midnight of the preferred day, study personnel contact the parent the following day (preferred day +1 business day) and remind them to complete the assessment or prompt their child to do so. If the participant does not access the system by midnight of the day following their preferred day, study personnel again attempt to contact the parent by phone (preferred day +2 business days) to remind them or prompt their child to access the system. Study personnel ascertain any issues that might be related to noncompliance (e.g., hospitalization) during reminder calls. "

"When a child's symptom score exceeds the pre-set severity threshold (i.e., scores 3 or higher), the system will generate an email alert through Epic messaging to the study team with the child's study ID. Upon receipt of an alert, the study coordinator will access the report in Epic and notify the child's treating team to determine whether a call is needed. If needed, the nurse will contact the parent within one business day to ascertain their child's current status (see Figure 3 for study schema). The nurse will document actions taken in Epic."

"After children and parents complete the week 8 and week 16 assessments, study personnel print out symptom reports (Group A: week 8 and week 16; Group B: week 16 only) in clinic and deliver them to parents of IP participants and treating provider prior to their appointment (either week-8 or week-16). Parents are also be encouraged to review the online version of the report by logging into MyChart prior to clinical visits. Clinicians in the child's care team are able to review this report in children's medical record, where it is stored along with other usual clinical data. These reports reflect children's cumulative symptom scores between baseline, week-8 and week-16 (Group A in Figure 2) or between week-8 to week-16 (Group B). Parents are encouraged to discuss the

5-ix) Describe use parameters

Describe use parameters (e.g., intended “doses” and optimal timing for use). Clarify what instructions or recommendations were given to the user, e.g., regarding timing, frequency, heaviness of use, if any, or was the intervention used ad libitum.

subitem not at all important

1 ◯

2 ◯

3 ◯

4 ◯

5 ◯

essential

Does your paper address subitem 5-ix?

Copy and paste relevant sections from the manuscript (include quotes in quotation marks "like this" to indicate direct quotes from your manuscript), or elaborate on this item by providing additional information not in the ms, or briefly explain why the item is not applicable/relevant for your study

Your answer

5-x) Clarify the level of human involvement

Clarify the level of human involvement (care providers or health professionals, also technical assistance) in the e-intervention or as co-intervention (detail number and expertise of professionals involved, if any, as well as "type of assistance offered, the timing and frequency of the support, how it is initiated, and the medium by which the assistance is delivered". It may be necessary to distinguish between the level of human involvement required for the trial, and the level of human involvement required for a routine application outside of a RCT setting (discuss under item 21 – generalizability).

subitem not at all important

1 ○

2 ○

3 ○

4 ○

5 ○

essential

Does your paper address subitem 5-x?

Copy and paste relevant sections from the manuscript (include quotes in quotation marks "like this" to indicate direct quotes from your manuscript), or elaborate on this item by providing additional information not in the ms, or briefly explain why the item is not applicable/relevant for your study

Your answer

5-xi) Report any prompts/reminders used

Report any prompts/reminders used: Clarify if there were prompts (letters, emails, phone calls, SMS) to use the application, what triggered them, frequency etc. It may be necessary to distinguish between the level of prompts/reminders required for the trial, and the level of prompts/reminders for a routine application outside of a RCT setting (discuss under item 21 – generalizability).

subitem not at all important

1 ○

2 ○

3 ○

4 ◉

5 ○

essential

Clear selection

Does your paper address subitem 5-xi? *

Copy and paste relevant sections from the manuscript (include quotes in quotation marks "like this" to indicate direct quotes from your manuscript), or elaborate on this item by providing additional information not in the ms, or briefly explain why the item is not applicable/relevant for your study

"IP participants are asked to choose a standard day of the week to access the system and complete their weekly symptom assessment. Parents of IP participants are informed that the study team will send a reminder to them one day prior to the chosen day regarding the upcoming assessment. IP participants are given a window of one business day before and two business days after their preferred day to access the system. IP participants complete the SyMon-SAYS symptom checklist (symptom items are available in Appendix) every week for 8 weeks through Epic MyChart via mobile app, computer or tablet. If IP participants do not access the system by midnight of the preferred day, study personnel contact the parent the following day (preferred day +1 business day) and remind them to complete the assessment or prompt their child to do so. If the participant does not access the system by midnight of the day following their preferred day, study personnel again attempt to contact the parent by phone (preferred day +2 business days) to remind them or prompt their child to access the system. "

5-xii) Describe any co-interventions (incl. training/support)

Describe any co-interventions (incl. training/support): Clearly state any interventions that are provided in addition to the targeted eHealth intervention, as ehealth intervention may not be designed as stand-alone intervention. This includes training sessions and support [1]. It may be necessary to distinguish between the level of training required for the trial, and the level of training for a routine application outside of a RCT setting (discuss under item 21 – generalizability.

subitem not at all important

1 ○

2 ○

3 ◉

4 ○

5 ○

essential

Clear selection

Does your paper address subitem 5-xii? *

Copy and paste relevant sections from the manuscript (include quotes in quotation marks "like this" to indicate direct quotes from your manuscript), or elaborate on this item by providing additional information not in the ms, or briefly explain why the item is not applicable/relevant for your study

"After enrollment, study personnel demonstrate how to complete baseline assessments using an iPad. IP participants are trained on accessing SyMon-SAYS using the Epic MyChart patient portal. IP participants are provided with a card containing their unique ID code and brief instructions for using the system. The card also contains the study personnel's name and telephone number for any questions that might arise.

6a) Completely defined pre-specified primary and secondary outcome measures, including how and when they were assessed

Does your paper address CONSORT subitem 6a? *

Copy and paste relevant sections from the manuscript (include quotes in quotation marks "like this" to indicate direct quotes from your manuscript), or elaborate on this item by providing additional information not in the ms, or briefly explain why the item is not applicable/relevant for your study

"For the quantitative methodology, we will calculate percentage of weekly symptom assessments that children complete, the number of weekly symptom scores that exceed a prior threshold to trigger alerts, as well as clinician documentation to symptom alerts in the EHR. In addition, a subset of participants (parent/child dyads) will be interviewed to obtain their feedback on the symptom report discussions with their clinician and their experience participating in the SyMon-SAYS study at the end of the intervention phase. Children and parents will be interviewed independently. Efforts will be made to balance gender (children and parents), ages (children), clinical variables of children as well as English- and Spanish-speaking participants (all children are English-speaking). Participants whose parents speak Spanish will be interviewed in Spanish.
At the completion of the study, clinicians, including attending physicians, fellows, advanced practice nurses/physician assistants, and registered nurses, will also be interviewed by either individual interviews (via telephone) or email survey to obtain their feedback on the symptom report, the study, and suggestions on how to make it more accessible/child friendly.

We plan to interview up to 30 parent/child dyads and up to 20 clinicians. The telephone interviews will be recorded for transcription and subsequent summary of feedback. Members of the study team will use selective qualitative analysis methods and an iterative coding process to identify common themes and develop coding rules to apply to interviewees' comments. The comments will then be compiled and summarized in frequency tables denoting the number of times certain responses emerge. Both frequency and the relative importance placed on the theme will be used evaluate usability, acceptability, and engagement along with quantitative results.

Adherence to Intervention
The adherence to the SyMon-SAYS Intervention will be evaluated by using the percentage of dyads completing the assessments at all time-points, excluding those who are off-study or deceased.

Measures used for Primary and Secondary Outcomes
Modified Symptom Management Barriers Questionnaire (SMBQ)
The modified SMBQ is a 23-item tool designed to assess the attitude (perceived barriers) of parents with children diagnosed with cancer towards the assessment and management of their child's symptoms. The SMBQ comprises items that encompass various recognized barriers to achieve effective symptom management. The original version of the SMBQ[9] items were devised with input from experts in symptom assessment and management and interviews with patients and their spouses. The study team made adjustments to the items to ensure their suitability for parents of children with cancer, such as replacing the pronoun "I" with "my child".

Pediatric PROMIS (pedsPROMIS)
Change in HRQOL over time will be analyzed by examining changes in the pediatric PROMIS measures. We will administer CATs of the domains in the PROMIS Pediatric Profile:[50] 1) physical function-mobility, 2) physical function-upper extremity, 3) depressive symptoms, 4) anxiety, 5) anger, and 6) relationship with peers. The pedsPROMIS offer good reliability across 2-3 standard deviations of the experiences of children 8-17 years old.

[illegible]

Self-Efficacy: NIH Toolbox Self-Efficacy
The NIH Toolbox initiative was developed to identify, create, and validate measures in the broad domains of cognitive function, emotional health, motor function, and sensory function.[51, 52] Measures of self-efficacy, defined as a person's belief in their capacity to manage their functioning and have control over meaningful events, were developed within the emotional health domain. This measure is available for children ages 8-12, adolescents aged 13-17 and adults aged 18+ and is available as CAT. These measures demonstrated good psychometric properties; US general population norms are available. Patients and parents will complete age appropriate CATs."

6a-i) Online questionnaires: describe if they were validated for online use and apply CHERRIES items to describe how the questionnaires were designed/deployed

If outcomes were obtained through online questionnaires, describe if they were validated for online use and apply CHERRIES items to describe how the questionnaires were designed/deployed [9].

subitem not at all important

1 ○

2 ○

3 ○

4 ○

5 ○

essential

Does your paper address subitem 6a-i?

Copy and paste relevant sections from manuscript text

Your answer

6a-ii) Describe whether and how “use” (including intensity of use/dosage) was defined/measured/monitored

Describe whether and how “use” (including intensity of use/dosage) was defined/measured/monitored (logins, logfile analysis, etc.). Use/adoption metrics are important process outcomes that should be reported in any ehealth trial.

subitem not at all important

1 ○

2 ○

3 ○

4 ○

5 ○

essential

Does your paper address subitem 6a-ii?

Copy and paste relevant sections from manuscript text

Your answer

6a-iii) Describe whether, how, and when qualitative feedback from participants was obtained

Describe whether, how, and when qualitative feedback from participants was obtained (e.g., through emails, feedback forms, interviews, focus groups).

subitem not at all important

1

2

3

4

5

essential

Does your paper address subitem 6a-iii?

Copy and paste relevant sections from manuscript text

Your answer

6b) Any changes to trial outcomes after the trial commenced, with reasons

Does your paper address CONSORT subitem 6b? *

Copy and paste relevant sections from the manuscript (include quotes in quotation marks "like this" to indicate direct quotes from your manuscript), or elaborate on this item by providing additional information not in the ms, or briefly explain why the item is not applicable/relevant for your study

There have been no changes to trial outcomes.

7a) How sample size was determined

NPT: When applicable, details of whether and how the clustering by care provides or centers was addressed

7a-i) Describe whether and how expected attrition was taken into account when calculating the sample size

Describe whether and how expected attrition was taken into account when calculating the sample size.

subitem not at all important

1 ○

2 ○

3 ○

4 ○

5 ○

essential

Does your paper address subitem 7a-i?

Copy and paste relevant sections from manuscript title (include quotes in quotation marks "like this" to indicate direct quotes from your manuscript), or elaborate on this item by providing additional information not in the ms, or briefly explain why the item is not applicable/relevant for your study

Your answer

7b) When applicable, explanation of any interim analyses and stopping guidelines

Does your paper address CONSORT subitem 7b? *

Copy and paste relevant sections from the manuscript (include quotes in quotation marks "like this" to indicate direct quotes from your manuscript), or elaborate on this item by providing additional information not in the ms, or briefly explain why the item is not applicable/relevant for your study

"The Data and Safety Monitoring Board (DSMB) of the study provides oversight for data and safety monitoring for this trial. The DSMB consists of three members outside of Northwestern University and Ann & Robert Lurie Children's Hospital of Chicago, who have extensive experience in research with clinical trials, patient-centered outcomes and pediatric oncology. DSMB and the study teams meets annually to review the data and any adverse events that occurred."

8a) Method used to generate the random allocation sequence

NPT: When applicable, how care providers were allocated to each trial group

Does your paper address CONSORT subitem 8a? *

Copy and paste relevant sections from the manuscript (include quotes in quotation marks "like this" to indicate direct quotes from your manuscript), or elaborate on this item by providing additional information not in the ms, or briefly explain why the item is not applicable/relevant for your study

"We will use a stratified block randomization approach. Participants are randomly assigned to Group A or B, stratified by gender, age, and cancer types. To achieve allocation concealment, the computer-based data management system (REDCap) assigns participants to one of two groups after the study enrollment procedures. Participants are informed of their group assignment (Group A or B) after the baseline

8b) Type of randomisation; details of any restriction (such as blocking and block size)

Does your paper address CONSORT subitem 8b? *

Copy and paste relevant sections from the manuscript (include quotes in quotation marks "like this" to indicate direct quotes from your manuscript), or elaborate on this item by providing additional information not in the ms, or briefly explain why the item is not applicable/relevant for your study

"We will use a stratified block randomization approach. Participants are randomly assigned to Group A or B, stratified by gender, age, and cancer types. To achieve allocation concealment, the computer-based data management system (REDCap) assigns participants to one of two groups after the study enrollment procedures. Participants are informed of their group assignment (Group A or B) after the baseline

9) Mechanism used to implement the random allocation sequence (such as sequentially numbered containers), describing any steps taken to conceal the sequence until interventions were assigned

Does your paper address CONSORT subitem 9? *

Copy and paste relevant sections from the manuscript (include quotes in quotation marks "like this" to indicate direct quotes from your manuscript), or elaborate on this item by providing additional information not in the ms, or briefly explain why the item is not applicable/relevant for your study

"We will use a stratified block randomization approach. Participants are randomly assigned to Group A or B, stratified by gender, age, and cancer types. To achieve allocation concealment, the computer-based data management system (REDCap) assigns participants to one of two groups after the study enrollment procedures. Participants are informed of their group assignment (Group A or B) after the baseline

10) Who generated the random allocation sequence, who enrolled participants, and who assigned participants to interventions

Does your paper address CONSORT subitem 10? *

Copy and paste relevant sections from the manuscript (include quotes in quotation marks "like this" to indicate direct quotes from your manuscript), or elaborate on this item by providing additional information not in the ms, or briefly explain why the item is not applicable/relevant for your study

Participants are enrolled by members of the study team.

"To achieve allocation concealment, the computer-based data management system (REDCap) assigns participants to one of two groups after the study enrollment procedures. Participants are informed of their group assignment (Group A or B) after the baseline assessment."

11a) If done, who was blinded after assignment to interventions (for example, participants, care providers, those assessing outcomes) and how

NPT: Whether or not administering co-interventions were blinded to group assignment

11a-i) Specify who was blinded, and who wasn't

Specify who was blinded, and who wasn't. Usually, in web-based trials it is not possible to blind the participants [1, 3] (this should be clearly acknowledged), but it may be possible to blind outcome assessors, those doing data analysis or those administering co-interventions (if any).

subitem not at all important

- 1 (selected)
- 2
- 3
- 4
- 5

essential

Clear selection

Does your paper address subitem 11a-i? *

Copy and paste relevant sections from the manuscript (include quotes in quotation marks "like this" to indicate direct quotes from your manuscript), or elaborate on this item by providing additional information not in the ms, or briefly explain why the item is not applicable/relevant for your study

Neither participants, clinical providers, nor members of the study team were blinded.

11a-ii) Discuss e.g., whether participants knew which intervention was the "intervention of interest" and which one was the "comparator"

Informed consent procedures (4a-ii) can create biases and certain expectations - discuss e.g., whether participants knew which intervention was the "intervention of interest" and which one was the "comparator".

subitem not at all important

1 ○

2 ○

3 ○

4 ○

5 ○

essential

Does your paper address subitem 11a-ii?

Copy and paste relevant sections from the manuscript (include quotes in quotation marks "like this" to indicate direct quotes from your manuscript), or elaborate on this item by providing additional information not in the ms, or briefly explain why the item is not applicable/relevant for your study

Your answer

11b) If relevant, description of the similarity of interventions
(this item is usually not relevant for ehealth trials as it refers to similarity of a placebo or sham intervention to a active medication/intervention)

Does your paper address CONSORT subitem 11b? *

Copy and paste relevant sections from the manuscript (include quotes in quotation marks "like this" to indicate direct quotes from your manuscript), or elaborate on this item by providing additional information not in the ms, or briefly explain why the item is not applicable/relevant for your study

"Participants (parents and children) complete assessments in-clinic using an iPad with internet access at baseline, week-8 and week-16. They are randomly assigned to Group A (weeks 1-16: SyMon-SAYS intervention) or Group B (weeks 1-8: usual care; weeks 9-16: SyMon-SAYS intervention) after the baseline assessment. During the intervention weeks (either weeks 1-16 or weeks 9-16), participants complete a weekly symptom assessment "

12a) Statistical methods used to compare groups for primary and secondary outcomes
NPT: When applicable, details of whether and how the clustering by care providers or centers was addressed

Does your paper address CONSORT subitem 12a? *

Copy and paste relevant sections from the manuscript (include quotes in quotation marks "like this" to indicate direct quotes from your manuscript), or elaborate on this item by providing additional information not in the ms, or briefly explain why the item is not applicable/relevant for your study

Evaluate efficacy of SyMon-SAYS after 8 weeks and its maintenance effects at week-16:
We will fit mixed effects models for repeated measures. We will control for multiplicity among co-primary outcomes with the Benjamini-Hochberg procedure.[53]
Regarding from baseline to week 8 (Group A) or from week 9 to week 16 (Group B) estimated by calculating the area under the curve (AUC) of each symptom score plotted over time for each participant. The AUC will be then divided by the total time to rescale back to the original units. We will calculate a p-value for differences in AUC curves between arms with a Wilcoxon rank test.

Evaluate predictors of adherence to the SyMon-SAYS intervention and preference of SyMon-SAYS versus usual care:
We will identify predictors of intervention adherence and preference using ordinal regression models using data from both groups' intervention phases (weeks 1-8 for group A, weeks 9-16 for group B). Based on number of weekly system assessments completed, patients will be classified as high adherence (completed 7 or 8 weekly assessments), intermediate adherence (completed 3-6 weekly assessments), or low adherence (completed 1 or 2 or fewer weekly assessments). Additionally, we will repeat the same analysis using data from week 9- week 16 from Group A to explore whether the group membership (i.e., high, intermediate or low adherence) changed between phase 1 (baseline to week 8) and phase 2 (week 9- week 16) as well as predictors associated with the group membership.

12a-i) Imputation techniques to deal with attrition / missing values

Imputation techniques to deal with attrition / missing values: Not all participants will use the intervention/comparator as intended and attrition is typically high in ehealth trials. Specify how participants who did not use the application or dropped out from the trial were treated in the statistical analysis (a complete case analysis is strongly discouraged, and simple imputation techniques such as LOCF may also be problematic [4]).

subitem not at all important

1 ○

2 ○

3 ◉

4 ○

5 ○

essential

Clear selection

Does your paper address subitem 12a-i? *

Copy and paste relevant sections from the manuscript (include quotes in quotation marks "like this" to indicate direct quotes from your manuscript), or elaborate on this item by providing additional information not in the ms, or briefly explain why the item is not applicable/relevant for your study

"If necessary, multiple imputation of weekly symptom data for secondary endpoints by chained equations (MICE) will be used to impute missing data."

12b) Methods for additional analyses, such as subgroup analyses and adjusted analyses

Does your paper address CONSORT subitem 12b? *

Copy and paste relevant sections from the manuscript (include quotes in quotation marks "like this" to indicate direct quotes from your manuscript), or elaborate on this item by providing additional information not in the ms, or briefly explain why the item is not applicable/relevant for your study

We will explore the associations of demographic variables (e.g., gender), individual factors (e.g., health literacy) and clinical characteristics (e.g., intensity of treatment) on primary outcomes.
We will code individual patients as having improved or not improved by a clinically important threshold and examine whether patient and parent contextual factors are associated with improvement on each outcome (each modeled separately) using multivariable logistic regression models.
"We will identify predictors of intervention adherence and preference using ordinal regression models using data from both groups' intervention phases (weeks 1-8 for group A, weeks 9-16 for group B). Based on number of weekly system assessments completed, patients will be classified as high adherence (completed 7 or 8 weekly assessments), intermediate adherence (completed 3-6 weekly assessments), or low adherence (completed 1 or 2 or fewer weekly assessments). "

X26) REB/IRB Approval and Ethical Considerations [recommended as subheading under "Methods"] (not a CONSORT item)

X26-i) Comment on ethics committee approval

subitem not at all important

1 ○

2 ○

3 ○

4 ○

5 ○

essential

Does your paper address subitem X26-i?

Copy and paste relevant sections from the manuscript (include quotes in quotation marks "like this" to indicate direct quotes from your manuscript), or elaborate on this item by providing additional information not in the ms, or briefly explain why the item is not applicable/relevant for your study

Your answer

x26-ii) Outline informed consent procedures

Outline informed consent procedures e.g., if consent was obtained offline or online (how? Checkbox, etc.?), and what information was provided (see 4a-ii). See [6] for some items to be included in informed consent documents.

subitem not at all important

1 ○

2 ○

3 ○

4 ○

5 ○

essential

Does your paper address subitem X26-ii?

Copy and paste relevant sections from the manuscript (include quotes in quotation marks "like this" to indicate direct quotes from your manuscript), or elaborate on this item by providing additional information not in the ms, or briefly explain why the item is not applicable/relevant for your study

Your answer

X26-iii) Safety and security procedures

Safety and security procedures, incl. privacy considerations, and any steps taken to reduce the likelihood or detection of harm (e.g., education and training, availability of a hotline)

subitem not at all important

1 ○

2 ○

3 ○

4 ○

5 ○

essential

Does your paper address subitem X26-iii?

Copy and paste relevant sections from the manuscript (include quotes in quotation marks "like this" to indicate direct quotes from your manuscript), or elaborate on this item by providing additional information not in the ms, or briefly explain why the item is not applicable/relevant for your study

Your answer

RESULTS

13a) For each group, the numbers of participants who were randomly assigned, received intended treatment, and were analysed for the primary outcome

NPT: The number of care providers or centers performing the intervention in each group and the number of patients treated by each care provider in each center

Does your paper address CONSORT subitem 13a? *

Copy and paste relevant sections from the manuscript (include quotes in quotation marks "like this" to indicate direct quotes from your manuscript), or elaborate on this item by providing additional information not in the ms, or briefly explain why the item is not applicable/relevant for your study

This is not applicable as enrollment is ongoing.

13b) For each group, losses and exclusions after randomisation, together with reasons

Does your paper address CONSORT subitem 13b? (NOTE: Preferably, this is shown in a CONSORT flow diagram) *

Copy and paste relevant sections from the manuscript (include quotes in quotation marks "like this" to indicate direct quotes from your manuscript), or elaborate on this item by providing additional information not in the ms, or briefly explain why the item is not applicable/relevant for your study

This is not applicable as enrollment is ongoing.

13b-i) Attrition diagram

Strongly recommended: An attrition diagram (e.g., proportion of participants still logging in or using the intervention/comparator in each group plotted over time, similar to a survival curve) or other figures or tables demonstrating usage/dose/engagement.

subitem not at all important

1 ○

2 ○

3 ○

4 ○

5 ○

essential

Does your paper address subitem 13b-i?

Copy and paste relevant sections from the manuscript or cite the figure number if applicable (include quotes in quotation marks "like this" to indicate direct quotes from your manuscript), or elaborate on this item by providing additional information not in the ms, or briefly explain why the item is not applicable/relevant for your study

Your answer

14a) Dates defining the periods of recruitment and follow-up

Does your paper address CONSORT subitem 14a? *

Copy and paste relevant sections from the manuscript (include quotes in quotation marks "like this" to indicate direct quotes from your manuscript), or elaborate on this item by providing additional information not in the ms, or briefly explain why the item is not applicable/relevant for your study

"Recruitment began in April 2021 and is expected to be completed in Spring, 2024. "

14a-i) Indicate if critical “secular events” fell into the study period

Indicate if critical “secular events” fell into the study period, e.g., significant changes in Internet resources available or “changes in computer hardware or Internet delivery resources”

subitem not at all important

1 ○

2 ○

3 ○

4 ○

5 ○

essential

Does your paper address subitem 14a-i?

Copy and paste relevant sections from the manuscript (include quotes in quotation marks "like this" to indicate direct quotes from your manuscript), or elaborate on this item by providing additional information not in the ms, or briefly explain why the item is not applicable/relevant for your study

Your answer

14b) Why the trial ended or was stopped (early)

Does your paper address CONSORT subitem 14b? *

Copy and paste relevant sections from the manuscript (include quotes in quotation marks "like this" to indicate direct quotes from your manuscript), or elaborate on this item by providing additional information not in the ms, or briefly explain why the item is not applicable/relevant for your study

This is not applicable because enrollment and data collection are ongoing.

15) A table showing baseline demographic and clinical characteristics for each group

NPT: When applicable, a description of care providers (case volume, qualification, expertise, etc.) and centers (volume) in each group

Does your paper address CONSORT subitem 15? *

Copy and paste relevant sections from the manuscript (include quotes in quotation marks "like this" to indicate direct quotes from your manuscript), or elaborate on this item by providing additional information not in the ms, or briefly explain why the item is not applicable/relevant for your study

This is not applicable because enrollment and data collection are ongoing.

15-i) Report demographics associated with digital divide issues

In ehealth trials it is particularly important to report demographics associated with digital divide issues, such as age, education, gender, social-economic status, computer/Internet/ehealth literacy of the participants, if known.

subitem not at all important

1 ○

2 ○

3 ○

4 ○

5 ○

essential

Does your paper address subitem 15-i? *

Copy and paste relevant sections from the manuscript (include quotes in quotation marks "like this" to indicate direct quotes from your manuscript), or elaborate on this item by providing additional information not in the ms, or briefly explain why the item is not applicable/relevant for your study

This is not applicable because enrollment and data collection are ongling.

16) For each group, number of participants (denominator) included in each analysis and whether the analysis was by original assigned groups

16-i) Report multiple “denominators” and provide definitions

Report multiple “denominators” and provide definitions: Report N’s (and effect sizes) “across a range of study participation [and use] thresholds” [1], e.g., N exposed, N consented, N used more than x times, N used more than y weeks, N participants “used” the intervention/comparator at specific pre-defined time points of interest (in absolute and relative numbers per group). Always clearly define “use” of the intervention.

subitem not at all important

1 ◯

2 ◯

3 ◯

4 ◯

5 ◯

essential

Does your paper address subitem 16-i? *

Copy and paste relevant sections from the manuscript (include quotes in quotation marks "like this" to indicate direct quotes from your manuscript), or elaborate on this item by providing additional information not in the ms, or briefly explain why the item is not applicable/relevant for your study

Not applicable. Enrollment and data collection are ongoing.

16-ii) Primary analysis should be intent-to-treat

Primary analysis should be intent-to-treat, secondary analyses could include comparing only “users”, with the appropriate caveats that this is no longer a randomized sample (see 18-i).

subitem not at all important

1

2

3

4

5

essential

Does your paper address subitem 16-ii?

Copy and paste relevant sections from the manuscript (include quotes in quotation marks "like this" to indicate direct quotes from your manuscript), or elaborate on this item by providing additional information not in the ms, or briefly explain why the item is not applicable/relevant for your study

Your answer

17a) For each primary and secondary outcome, results for each group, and the estimated effect size and its precision (such as 95% confidence interval)

Does your paper address CONSORT subitem 17a? *

Copy and paste relevant sections from the manuscript (include quotes in quotation marks "like this" to indicate direct quotes from your manuscript), or elaborate on this item by providing additional information not in the ms, or briefly explain why the item is not applicable/relevant for your study

Not applicable. Enrollment and data collection are ongoing.

17a-i) Presentation of process outcomes such as metrics of use and intensity of use

In addition to primary/secondary (clinical) outcomes, the presentation of process outcomes such as metrics of use and intensity of use (dose, exposure) and their operational definitions is critical. This does not only refer to metrics of attrition (13-b) (often a binary variable), but also to more continuous exposure metrics such as “average session length”. These must be accompanied by a technical description how a metric like a “session” is defined (e.g., timeout after idle time) [1] (report under item 6a).

subitem not at all important

1 ○

2 ○

3 ○

4 ○

5 ○

essential

Does your paper address subitem 17a-i?

Copy and paste relevant sections from the manuscript (include quotes in quotation marks "like this" to indicate direct quotes from your manuscript), or elaborate on this item by providing additional information not in the ms, or briefly explain why the item is not applicable/relevant for your study

Your answer

17b) For binary outcomes, presentation of both absolute and relative effect sizes is recommended

Does your paper address CONSORT subitem 17b? *

Copy and paste relevant sections from the manuscript (include quotes in quotation marks "like this" to indicate direct quotes from your manuscript), or elaborate on this item by providing additional information not in the ms, or briefly explain why the item is not applicable/relevant for your study

Not applicable. Enrollment and data collection are ongoing.

18) Results of any other analyses performed, including subgroup analyses and adjusted analyses, distinguishing pre-specified from exploratory

Does your paper address CONSORT subitem 18? *

Copy and paste relevant sections from the manuscript (include quotes in quotation marks "like this" to indicate direct quotes from your manuscript), or elaborate on this item by providing additional information not in the ms, or briefly explain why the item is not applicable/relevant for your study

Not applicable. Enrollment and data collection are ongoing.

18-i) Subgroup analysis of comparing only users

A subgroup analysis of comparing only users is not uncommon in ehealth trials, but if done, it must be stressed that this is a self-selected sample and no longer an unbiased sample from a randomized trial (see 16-iii).

subitem not at all important

1 ○

2 ○

3 ○

4 ○

5 ○

essential

Does your paper address subitem 18-i?

Copy and paste relevant sections from the manuscript (include quotes in quotation marks "like this" to indicate direct quotes from your manuscript), or elaborate on this item by providing additional information not in the ms, or briefly explain why the item is not applicable/relevant for your study

Your answer

19) All important harms or unintended effects in each group

(for specific guidance see CONSORT for harms)

Does your paper address CONSORT subitem 19? *

Copy and paste relevant sections from the manuscript (include quotes in quotation marks "like this" to indicate direct quotes from your manuscript), or elaborate on this item by providing additional information not in the ms, or briefly explain why the item is not applicable/relevant for your study

Not applicable. Enrollment and data collection are ongoing.

19-i) Include privacy breaches, technical problems

Include privacy breaches, technical problems. This does not only include physical “harm” to participants, but also incidents such as perceived or real privacy breaches [1], technical problems, and other unexpected/unintended incidents. “Unintended effects” also includes unintended positive effects [2].

subitem not at all important

1 ○

2 ○

3 ○

4 ○

5 ○

essential

Does your paper address subitem 19-i?

Copy and paste relevant sections from the manuscript (include quotes in quotation marks "like this" to indicate direct quotes from your manuscript), or elaborate on this item by providing additional information not in the ms, or briefly explain why the item is not applicable/relevant for your study

Your answer

19-ii) Include qualitative feedback from participants or observations from staff/researchers

Include qualitative feedback from participants or observations from staff/researchers, if available, on strengths and shortcomings of the application, especially if they point to unintended/unexpected effects or uses. This includes (if available) reasons for why people did or did not use the application as intended by the developers.

subitem not at all important

1 ○

2 ○

3 ○

4 ○

5 ○

essential

Does your paper address subitem 19-ii?

Copy and paste relevant sections from the manuscript (include quotes in quotation marks "like this" to indicate direct quotes from your manuscript), or elaborate on this item by providing additional information not in the ms, or briefly explain why the item is not applicable/relevant for your study

Your answer

DISCUSSION

22) Interpretation consistent with results, balancing benefits and harms, and considering other relevant evidence

NPT: In addition, take into account the choice of the comparator, lack of or partial blinding, and unequal expertise of care providers or centers in each group

22-i) Restate study questions and summarize the answers suggested by the data, starting with primary outcomes and process outcomes (use)

Restate study questions and summarize the answers suggested by the data, starting with primary outcomes and process outcomes (use).

subitem not at all important

1 ○

2 ○

3 ○

4 ○

5 ○

essential

Does your paper address subitem 22-i? *

Copy and paste relevant sections from the manuscript (include quotes in quotation marks "like this" to indicate direct quotes from your manuscript), or elaborate on this item by providing additional information not in the ms, or briefly explain why the item is not applicable/relevant for your study

Study questions are summarized but results are not reported because enrollment and data collection are ongoing.

22-ii) Highlight unanswered new questions, suggest future research

Highlight unanswered new questions, suggest future research.

subitem not at all important

1 ○

2 ○

3 ○

4 ○

5 ○

essential

Does your paper address subitem 22-ii?

Copy and paste relevant sections from the manuscript (include quotes in quotation marks "like this" to indicate direct quotes from your manuscript), or elaborate on this item by providing additional information not in the ms, or briefly explain why the item is not applicable/relevant for your study

Your answer

20) Trial limitations, addressing sources of potential bias, imprecision, and, if relevant, multiplicity of analyses

20-i) Typical limitations in ehealth trials

Typical limitations in ehealth trials: Participants in ehealth trials are rarely blinded. Ehealth trials often look at a multiplicity of outcomes, increasing risk for a Type I error. Discuss biases due to non-use of the intervention/usability issues, biases through informed consent procedures, unexpected events.

subitem not at all important

1 ○

2 ○

3 ○

4 ○

5 ○

essential

Does your paper address subitem 20-i? *

Copy and paste relevant sections from the manuscript (include quotes in quotation marks "like this" to indicate direct quotes from your manuscript), or elaborate on this item by providing additional information not in the ms, or briefly explain why the item is not applicable/relevant for your study

"The study has some potential caveats that will need to be considered in interpreting the findings and should be addressed in future research. "

21) Generalisability (external validity, applicability) of the trial findings

NPT: External validity of the trial findings according to the intervention, comparators, patients, and care providers or centers involved in the trial

21-i) Generalizability to other populations

Generalizability to other populations: In particular, discuss generalizability to a general Internet population, outside of a RCT setting, and general patient population, including applicability of the study results for other organizations

subitem not at all important

1 ○

2 ○

3 ○

4 ○

5 ○

essential

Does your paper address subitem 21-i?

Copy and paste relevant sections from the manuscript (include quotes in quotation marks "like this" to indicate direct quotes from your manuscript), or elaborate on this item by providing additional information not in the ms, or briefly explain why the item is not applicable/relevant for your study

Your answer

21-ii) Discuss if there were elements in the RCT that would be different in a routine application setting

Discuss if there were elements in the RCT that would be different in a routine application setting (e.g., prompts/reminders, more human involvement, training sessions or other co-interventions) and what impact the omission of these elements could have on use, adoption, or outcomes if the intervention is applied outside of a RCT setting.

subitem not at all important

1 ◯

2 ◯

3 ◯

4 ◯

5 ◯

essential

Does your paper address subitem 21-ii?

Copy and paste relevant sections from the manuscript (include quotes in quotation marks "like this" to indicate direct quotes from your manuscript), or elaborate on this item by providing additional information not in the ms, or briefly explain why the item is not applicable/relevant for your study

Your answer

OTHER INFORMATION

23) Registration number and name of trial registry

Does your paper address CONSORT subitem 23? *

Copy and paste relevant sections from the manuscript (include quotes in quotation marks "like this" to indicate direct quotes from your manuscript), or elaborate on this item by providing additional information not in the ms, or briefly explain why the item is not applicable/relevant for your study

ClinicalTRials.gov Identifier: NCT04789720

24) Where the full trial protocol can be accessed, if available

Does your paper address CONSORT subitem 24? *

Cite a Multimedia Appendix, other reference, or copy and paste relevant sections from the manuscript (include quotes in quotation marks "like this" to indicate direct quotes from your manuscript), or elaborate on this item by providing additional information not in the ms, or briefly explain why the item is not applicable/relevant for your study

The full protocol is not currently available.

25) Sources of funding and other support (such as supply of drugs), role of funders

Does your paper address CONSORT subitem 25? *

Copy and paste relevant sections from the manuscript (include quotes in quotation marks "like this" to indicate direct quotes from your manuscript), or elaborate on this item by providing additional information not in the ms, or briefly explain why the item is not applicable/relevant for your study

This trial is funded by a cooperative agreement with the National Cancer Institute (5U01 CA246612; PI: Jin-Shei Lai). The funders had no role in study design, data collection and analysis, decision to publish, or preparation of the manuscript.

X27) Conflicts of Interest (not a CONSORT item)

X27-i) State the relation of the study team towards the system being evaluated

In addition to the usual declaration of interests (financial or otherwise), also state the relation of the study team towards the system being evaluated, i.e., state if the authors/evaluators are distinct from or identical with the developers/sponsors of the intervention.

subitem not at all important

1 ○

2 ○

3 ◉

4 ○

5 ○

essential

Clear selection

Does your paper address subitem X27-i?

Copy and paste relevant sections from the manuscript (include quotes in quotation marks "like this" to indicate direct quotes from your manuscript), or elaborate on this item by providing additional information not in the ms, or briefly explain why the item is not applicable/relevant for your study

The authors having nothing to disclose.

About the CONSORT EHEALTH checklist

As a result of using this checklist, did you make changes in your manuscript? *

- ( ) yes, major changes
- ( ) yes, minor changes
- (•) no

What were the most important changes you made as a result of using this checklist?

Your answer

How much time did you spend on going through the checklist INCLUDING making changes in your manuscript *

Several hours going through the checklist. No manuscript changes were made as a

As a result of using this checklist, do you think your manuscript has improved? *

- ( ) yes
- (•) no
- ( ) Other:

Would you like to become involved in the CONSORT EHEALTH group?

This would involve for example becoming involved in participating in a workshop and writing an "Explanation and Elaboration" document

- ○ yes
- ◉ no
- ○ Other:

Clear selection

Any other comments or questions on CONSORT EHEALTH

Your answer

STOP - Save this form as PDF before you click submit

To generate a record that you filled in this form, we recommend to generate a PDF of this page (on a Mac, simply select "print" and then select "print as PDF") before you submit it.

When you submit your (revised) paper to JMIR, please upload the PDF as supplementary file.

Don't worry if some text in the textboxes is cut off, as we still have the complete information in our database. Thank you!

Final step: Click submit !

Click submit so we have your answers in our database!

Submit Clear form

Never submit passwords through Google Forms.

This content is neither created nor endorsed by Google. Report Abuse - Terms of Service - Privacy Policy

Google Forms
